# Single-Cell Transcriptomics Identifies Heterogeneity of Mouse Mammary Gland Fibroblasts With Distinct Functions, Estrogen Responses, Differentiation Processes, and Crosstalks With Epithelium

**DOI:** 10.3389/fcell.2022.850568

**Published:** 2022-03-01

**Authors:** Ryohei Yoshitake, Gregory Chang, Kohei Saeki, Desiree Ha, Xiwei Wu, Jinhui Wang, Shiuan Chen

**Affiliations:** ^1^ Department of Cancer Biology, Beckman Research Institute of City of Hope, Duarte, CA, United States; ^2^ Faculty of Veterinary Medicine, Okayama University of Science, Imabari, Japan; ^3^ Integrative Genomics Core, Beckman Research Institute of City of Hope, Monrovia, CA, United States

**Keywords:** cell-cell interaction, estrogen, fibroblasts, mammary gland, single-cell RNA sequencing, trajectory analysis

## Abstract

Fibroblasts have been shown to be one of the essential players for mammary gland organization. Here, we identify two major types of mouse mammary gland fibroblasts through single-cell RNA sequencing analysis: *Dpp4* ^*+*^ fibroblasts and *Dpp4* ^*-*^ fibroblasts. Each population exhibits unique functional characteristics as well as discrete localization in normal mouse mammary glands. Remarkably, estrogen, a crucial mediator of mammary gland organization, alters the gene expression profiles of fibroblasts in a population-specific manner, without distinct activation of estrogen receptor signaling. Further integrative analysis with the inclusion of five other publicly available datasets reveals a directional differentiation among the mammary gland fibroblast populations. Moreover, the combination with the mouse mammary epithelium atlas allows us to infer multiple potential interactions between epithelial cells and fibroblasts in mammary glands. This study provides a comprehensive view of mouse mammary gland fibroblasts at the single-cell level.

## Introduction

The mammary gland is a dynamic organ that undergoes major morphological changes after birth to develop its fully functional structure and is continuously modulated by ovarian hormones, such as estrogen and progesterone, with cyclic changes in their levels. The mammary gland is comprised of two major components: mammary epithelium, which forms mammary ductal and lobular structures, and mammary stroma, which includes fibroblasts, adipocytes, preadipocytes, endothelial cells, and/or immune cells. Although the epithelium has been recognized to be an essential player in the mammary gland, the involvement of fibroblasts in mammary gland organization has also been extensively discussed (Wiseman and Werb, 2002; Sumbal et al., 2021). For instance, fibroblasts are important for the production and remodeling of extracellular matrix (ECM). Since ECM undergoes significant modifications during mammary gland organization such as ductal elongation and branching morphogenesis, fibroblasts are considered essential for proper gland development (Maller et al., 2010; Schedin and Keely, 2011). Also, epithelial-fibroblast crosstalk through a number of mediators has been shown to be critical in mammary gland development as well as maintenance. Thus, mammary gland fibroblasts contribute towards many aspects of mammary gland organization, and unfortunately, carcinogenesis as well.

Mouse mammary glands have been utilized as important models for studying mammary gland biology such as development, homeostasis under the influence of ovarian hormones, and/or pathological processes. Although there are structural differences between human and mouse mammary glands, such as the lack of terminal duct lobular units (TDLUs) and the enrichment of adipose tissues in mouse glands, both human and mouse mammary glands share many similarities in epithelial cell features and developmental processes. While the population-specific roles of mammary gland fibroblasts in human breast tissues are just beginning to be unraveled (Morsing et al., 2016; Morsing et al., 2020), the characterization of mouse mammary gland fibroblasts would help to deepen the understanding of the mammary gland in both humans and mice. The functional roles of mammary gland fibroblasts have been accumulated using mouse models (Wiseman and Werb, 2002; Sumbal et al., 2021) and then extrapolated to human breast studies. Although the fibroblast populations in the mouse mammary gland have been shown to be heterogeneous (Sumbal et al., 2021), their detailed properties and contributions to mammary gland organization remains inadequately defined.

Recent development of single-cell RNA sequencing (scRNA-seq) technology allows us to investigate gene expression profiles of whole cellular populations in a given organ or tissue. Our group previously explored all the cell populations of the mouse mammary gland and elucidated their response to estrogen at a single-cell level (Kanaya et al., 2019). Moreover, in our recent publication, we established a mammary epithelial atlas by combining our own dataset with other public datasets, revealing the putative trajectory of mammary epithelium with different linages (Saeki et al., 2021).

Accordingly, the purpose of the current study is to comprehensively describe the heterogeneity of fibroblasts in mouse mammary gland in terms of their functions, localizations, differentiations, and interactions with the mammary epithelium. To this goal, we performed a nonbiased single-cell transcriptome analysis using scRNA-seq on mouse mammary gland fibroblasts, specifically using models mimicking menopausal transition in humans. We identified two major populations of mouse mammary gland fibroblasts, described as either *Dpp4* ^*+*^ or *Dpp4* ^*-*^ fibroblasts. Gene signature analysis revealed the differences in functions of the two types of fibroblasts for the organization of the mammary gland tissues. Histological evaluation showed the distinct localization of the two major fibroblast types in normal mouse mammary gland. Moreover, since our experiments were performed on hormone-depleted mouse models followed with hormone treatments, we could profile the population-specific effects of ovarian hormones, especially estrogen, on mouse mammary gland fibroblasts. Further integrative analyses including datasets from other investigators predicted the uniqueness in differentiation trajectory of mammary gland fibroblasts among various organs’ fibroblasts, and eventually, the potential epithelial-fibroblast cell interactions within mammary gland tissue.

## Results

### Mouse Mammary Gland in Two Different Experimental Models Showed Similar Phenotypical Changes in the Absence and Presence of Ovarian Hormones

Menopausal transition has been recognized as a window of susceptibility in a woman’s life because significant structural and functional changes occur in the mammary gland, as well as alterations in the mammary micro-environment and hormone signaling, that may influence breast cancer risk (Terry et al., 2019). To properly examine the effects of estrogen and progesterone on the mouse mammary gland, resembling menopausal transition in women, we firstly evaluated phenotypical changes in two different ovarian hormone-depleted mouse models: ovariectomy (OVX; a surgical menopausal model) (Saeki et al., 2021) and 4-vinylcyclohexene diepoxide (VCD) models (Supplementary Figure S1A). Briefly, in the OVX model, ovaries of Balb/cJ mice were surgically removed bilaterally and then the mice were treated with either vehicle, 17β-estradiol (E2), or E2 + progesterone (P4) (Saeki et al., 2021). In the more recent VCD menopausal transition model, VCD’s ovarian toxicity gradually destroyed primordial and primary follicles, therefore accomplishing menopause in C57BL/6J mice through the deletion of ovarian hormones. After complete ovarian failure, for determining the impact of the exposure of E2 and P4, the mice were treated with vehicle, E2, E2 + P4, or E2 + ICI 182,780 (ICI). ICI is an estrogen receptor (ER) degrader which eliminates E2 action. For the VCD model, mice that did not undergo VCD treatment were included as the intact group. In the phenotypical analysis on whole mount staining (Supplementary Figure S1B), the regression of mammary ductal structures was observed in the vehicle groups from both the OVX and VCD models. E2 as well as E2 + P4 groups showed the ductal regrowth and the formation of terminal end bud-like structures at the tip of the duct in both models. E2 + ICI treatment in the VCD model showed sparse ductal structures compared to that of the E2- or E2 + P4-treated mice, confirming active E2-ER signaling during the ductal regrowth in mammary glands. Since both the OVX and VCD models showed comparable phenotypes in response to the depletion and administration of the hormones, we decided to integrate the scRNA-seq data from these two treatment models to investigate mouse mammary gland fibroblasts.

### Single-Cell Transcriptome Analyses Identified Two Major Types of Mammary Gland Fibroblasts With Distinct Gene Signatures and Localizations as *Dpp4* ^*+*^ and *Dpp4* ^*-*^ Fibroblasts

**FIGURE 1:**
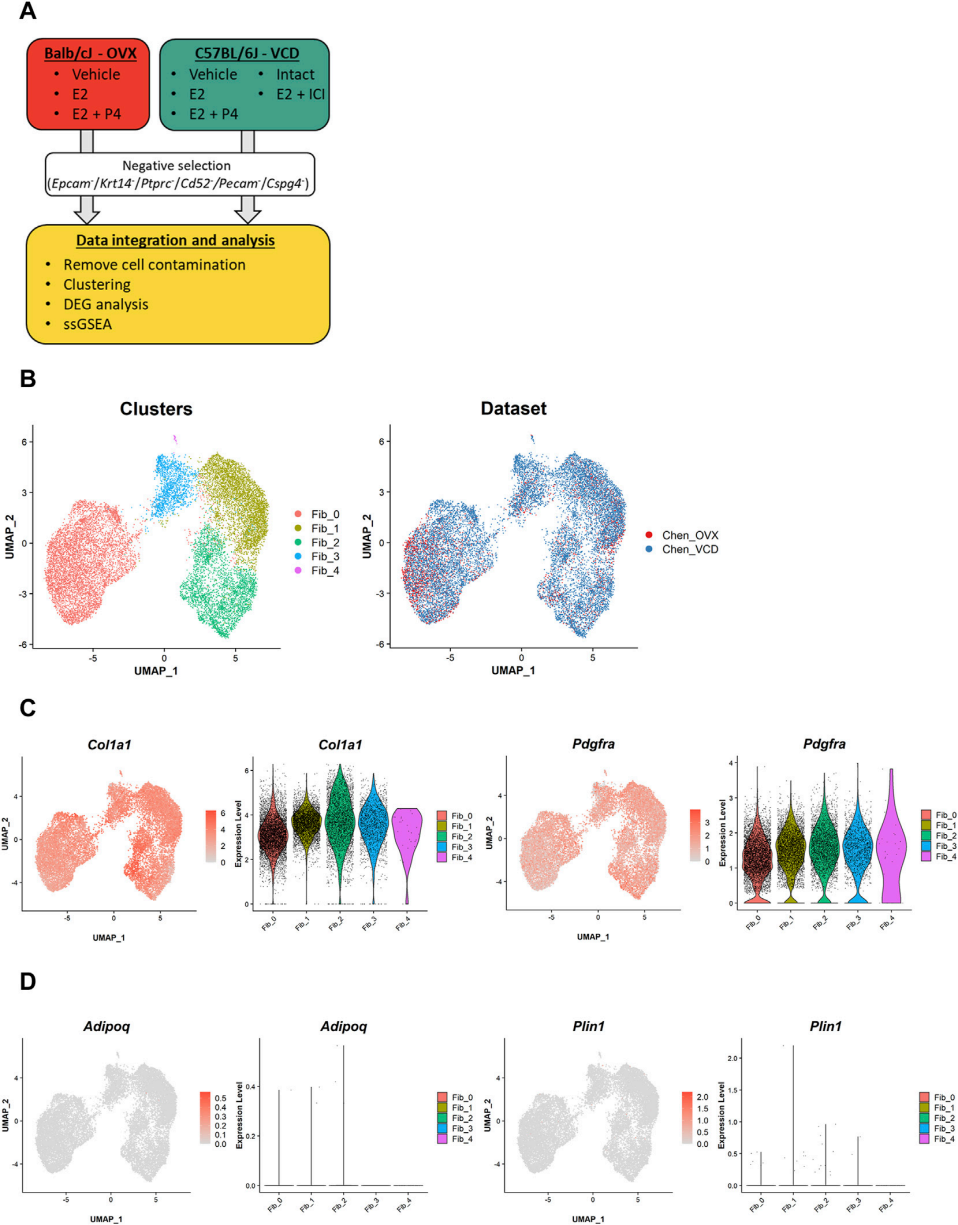
Our scRNA-seq datasets identify two major clusters of mammary gland fibroblasts commonly existing in the two different mouse models. **(A)** The schematic diagram of data processing and analyses for our own scRNA-seq datasets from the OVX and VCD models. The single-cell data were corrected from the mammary glands of the two models. Then, mammary gland fibroblasts were sorted by negative selection (i.e., sorting for cells without read counts for *Epcam*, *Krt14*, *Ptprc*, *Cd52*, *Pecam1*, and *Cspg4*). After the additional removal of a small fraction of cell contamination from other lineages of cells, the fibroblast dataset was integrated and evaluated in the downstream analyses (i.e., clustering, DEG analysis, and ssGSEA scoring). **(B)** The UMAP plot of the integrated dataset. Each cell is represented by a dot (*n* = 16,197) and is colored according to the cluster identified by the unbiased clustering (left) and the originated datasets (right). **(C)** The expression of the fibroblast markers (*Col1a1* and *Pdgfra*) and **(D)** the mature adipocyte markers (*Adipoq* and *Plin1*). Each feature plot on the left panel shows the distribution of the marker-expressing cells with color indicating the expression level. Each violin plot on the right panel shows the expression level of each gene in each cluster.

To profile fibroblasts in the mouse mammary gland, we first sorted fibroblasts using negative selection (i.e., sorting for cells without read counts for *Epcam*, *Krt14*, *Ptprc*, *Cd52*, *Pecam1*, and *Cspg4*) as described in previous studies (Figure 1A) (Bartoschek et al., 2018; Kanaya et al., 2019). Then, we integrated the data from both the OVX and VCD models using the anchor-based method implemented in the Seurat R package. After further removal of a small fraction of cell contamination from other lineages, a total of 16,197 cells with an average of 3,229 genes and 14,774 UMI counts per cell were evaluated in the downstream analyses. In the Uniform Manifold Approximation and Projection (UMAP) plot, two major cell clusters and a minor cluster were identified (Figure 1B, left). The major cluster on the right of the UMAP plot consisted of three subclusters [fibroblast cluster 1–3 (Fib_1–3)]. Of note, the cells from the two different models were well distributed throughout each cluster (Figure 1B, right), indicating that each cell population was commonly present in both the OVX and VCD models, independent of their mouse strains and different ovarian suppression protocols.

To ensure that we successfully sorted the mouse mammary gland fibroblasts, we evaluated marker gene expressions. The results showed that almost all the cells were positive for fibroblast markers (*Col1a1*, *Pdgfra*) (Figure 1C). The evaluation of the dataset including all isolated single cells further confirmed the successful selection of mammary gland fibroblasts into our “fibroblast” dataset (Supplementary Figures S2A–C). Because we physically excluded larger cells during the single-cell dissociation process, almost all mature adipocytes represented by *Adipoq*/*Plin1* expression were removed, further ensuring the purity of our fibroblast dataset (Figure 1D).

**FIGURE 2:**
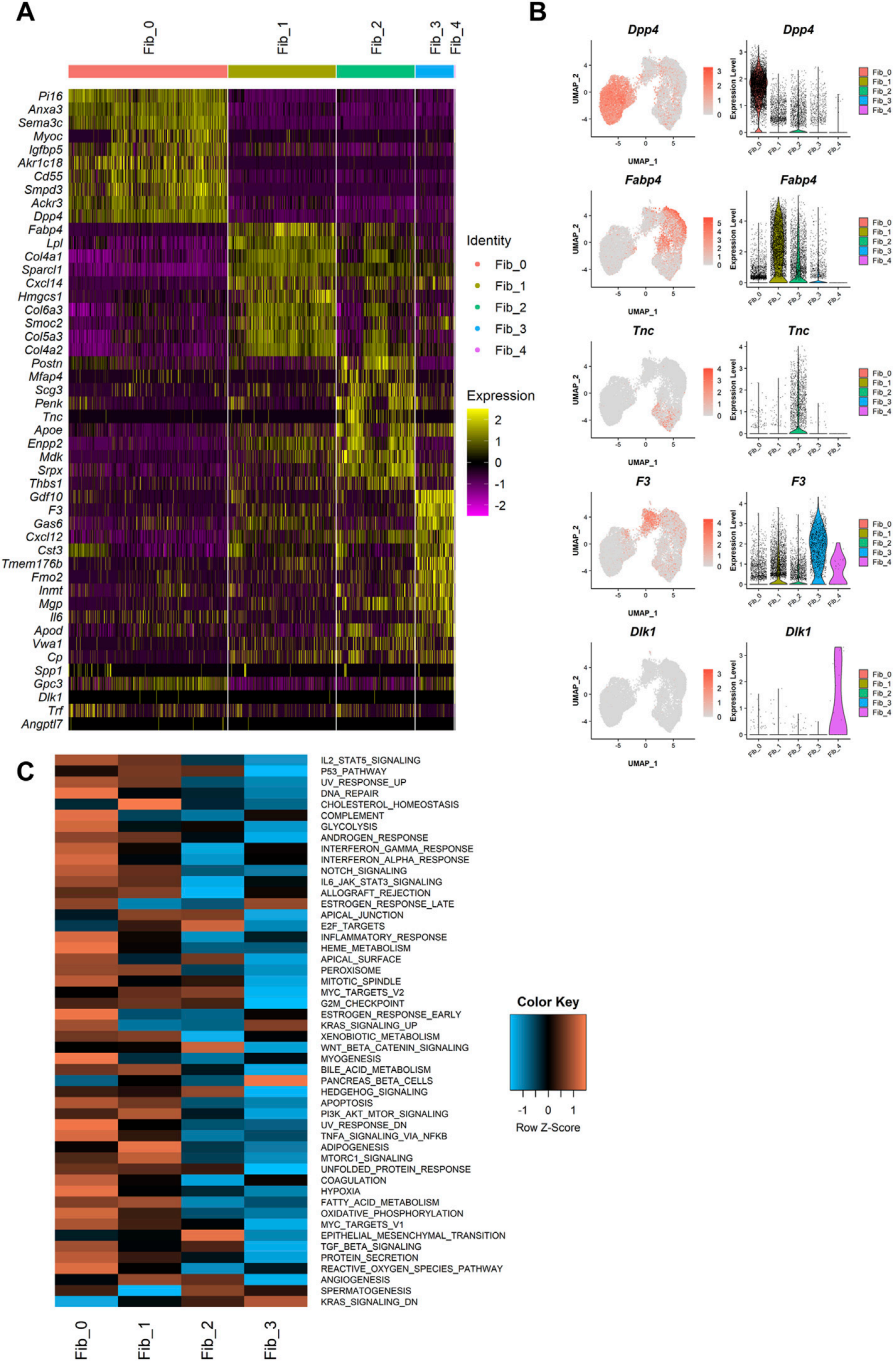
Characterization of the mammary gland fibroblast populations identified by the unbiased clustering. **(A)** The heatmap of the top 10 DEGs for each cluster. Each column represents each cell and each row represents each gene. Log-normalized gene count was scaled per gene. Color bar at the top indicates the cell clusters identified in Figure 1B. **(B)** The expressions of the identified marker genes for each cluster. Each feature plot on the left panel shows the distribution of the marker-expressing cells with color indicating the expression level. Each violin plot on the right panel shows the expression level of each gene in each cluster. **(C)** The heatmap of average ssGSEA scores for the hallmark gene sets in each cluster. ssGSEA score for each gene set was calculated from the log-normalized gene counts in each cell using the GSVA R package. Each row represents each gene set and each column represents the mean ssGSEA score for each cluster in each column. Color indicates the Z-score calculated by row.

Next, we performed differential expression testing using the Seurat package to detect the differentially expressed genes (DEGs) in each population (Figures 2A,B). One of the major clusters, Fib_0, showed highly specific gene expressions of *Dpp4*, *Pi16*, and *Anxa3*. Importantly, DPP4 is known to be a marker for one of the fibroblast subtypes in human breast tissue localizing outside of the TDLUs, called interlobular fibroblasts (Atherton et al., 1992; Morsing et al., 2016). Although mouse mammary glands do not have TDLU structures, the specific expression of *Dpp4* suggested the similarity of the *Dpp4* ^*+*^ Fib_0 fibroblasts in mouse mammary glands to the human interlobular fibroblasts. In the other major cluster, consisting of *Dpp4* ^*-*^ fibroblasts with three subclusters, Fib_1 cells showed higher expressions of adipogenic cell markers, *Fabp4* and *Lpl*, suggesting their commitment to adipogenesis. Fib_2 cells were characterized by the high expressions of ECM genes such as *Postn*, *Mfap4*, and *Tnc*. Fib_3 cells were positive for *Gdf10* and *F3*, which were identified to be the markers for “adipo-regulatory cells” observed in mouse white adipose tissues and skeletal muscle in recent studies (Schwalie et al., 2018; Camps et al., 2020). A minor cluster, Fib_4, had an explicit expression of the preadipocyte marker, *Dlk1*, suggesting that Fib_4 fibroblasts represented the preadipocyte population in this dataset.

Then, to further profile the functional commitments of each population to the organization of mouse mammary gland, we performed a gene signature analysis using single-sample gene set enrichment analysis (ssGSEA) (Barbie et al., 2009; Hänzelmann et al., 2013). The ssGSEA calculates a score that summarizes the expression of a set of genes at a single-cell level (i.e., ssGSEA score). For this analysis, “hallmark” gene sets, which represent 50 well-defined and essential biological states or processes, were referred from Molecular Signature Database (MSigDB) (Liberzon et al., 2015). Fib_4 cells were removed from this analysis because of the small number of cells and their expression of preadipocyte gene features. The ssGSEA score for each gene set was visualized on a heatmap (Figure 2C), and the top five significant gene sets for each population were listed in Table 1. *Dpp4* ^*+*^ Fib_0 fibroblasts showed the enrichment of “INFLAMMATORY_RESPONSE.” Fib_1 cells showed upregulation of adipose-related gene sets such as “CHOLESTEROL_HOMEOSTASIS” and “ADIPOGENESIS,” further indicating that they are related to the organization of adipose tissue around the mammary gland ducts. Fib_2 cells had a higher score in “EPITHELIAL_MESENCHYMAL_TRANSITION,” which includes many ECM-related genes, suggesting their roles in the regulation of ECM within the mammary gland stroma. Fib_3 cells showed relatively low scores for the hallmark gene sets, suggesting lower activity compared to the Fib_0 to Fib_2 populations. These results were further supported by an enrichment analysis available on MSigDB (Liberzon et al., 2015) in which we computed overlaps between the DEGs of each population and the genes included in the hallmark gene sets (Supplementary Table S1). Importantly, although the ssGSEA scores for “ESTROGEN_RESPONSE_EARLY” and “ESTROGEN_RESPONSE_LATE” were suggested to be relatively higher in Fib_0 and Fib_3, respectively (Table 1), the list of top 5 upregulated gene signatures from the latter enrichment analysis on the highly upregulated genes in Fib_0 and Fib_3 did not include these estrogen-regulated gene sets (Supplementary Table S1). These results indicated that the overall activation of “typical” estrogen-regulated genes was less significant in mouse mammary gland fibroblasts, even in the Fib_0 and Fib_3 cells. Together, our scRNA-seq analysis revealed the potential roles of each fibroblast population in the mouse mammary gland stroma.

**TABLE 1:** Top 5 significant gene signatures and mean ssGSEA scores for each fibroblast population.

Pathway	Mean (within cluster)	Mean (the other clusters)	Adjusted *p* value
Fib_0			
ESTROGEN_RESPONSE_EARLY	0.198	0.174	0
INFLAMMATORY_RESPONSE	0.221	0.198	0
KRAS_SIGNALING_UP	0.190	0.173	0
INTERFERON_ALPHA_RESPONSE	0.328	0.273	5.9E-275
INTERFERON_GAMMA_RESPONSE	0.327	0.286	1.5E-256
Fib_1			
CHOLESTEROL_HOMEOSTASIS	0.332	0.283	6.4E-268
ADIPOGENESIS	0.387	0.365	8.7E-106
MTORC1_SIGNALING	0.381	0.359	3.4E-90
APICAL_JUNCTION	0.204	0.195	7.2E-63
ALLOGRAFT_REJECTION	0.201	0.195	3.7E-43
Fib_2			
EPITHELIAL_MESENCHYMAL_TRANSITION	0.494	0.464	1.4E-145
KRAS_SIGNALING_DN	−0.106	−0.112	2.5E-32
APICAL_JUNCTION	0.204	0.196	2.6E-22
E2F_TARGETS	0.192	0.187	3.0E-21
WNT_BETA_CATENIN_SIGNALING	0.168	0.156	1.9E-18
Fib_3			
KRAS_SIGNALING_DN	−0.101	−0.112	2.5E-37
KRAS_SIGNALING_UP	0.188	0.179	4.5E-29
ESTROGEN_RESPONSE_LATE	0.197	0.191	4.5E-16
PANCREAS_BETA_CELLS	0.129	0.126	0.00025
COAGULATION	0.336	0.338	0.268,562

**FIGURE 3:**
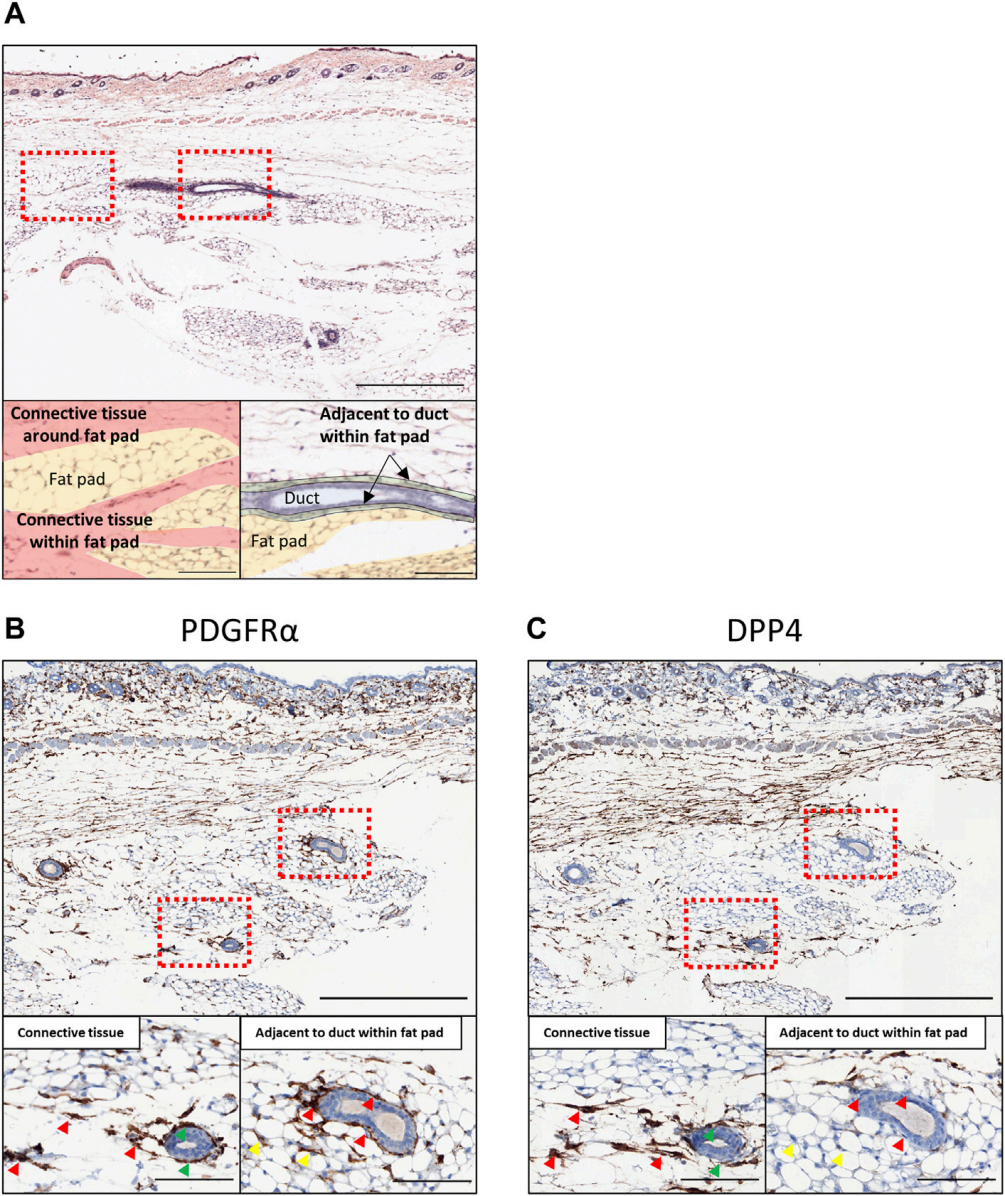
Histological evaluation in normal mouse mammary gland tissues. **(A)** The H&E staining of the normal mouse mammary gland. The bottom panels are colored according to histological annotation as indicated. **(B,C)** The immunohistochemical staining of normal mouse mammary gland for **(B)** PDGFRα and **(C)** DPP4. The mammary glands of eight-week-old C57BL/6J mice were collected together with skin (at the top part of the images) and cross-sectioned. The upper panels show the entire images of the mammary glands at low magnifications. The lower panels show the magnified images of the boxed area for the connective tissue region around/within the mammary fat pad (left) and the region adjacent to mammary gland ducts within the fat pad (right). Red arrowheads indicate the representative staining pattern of each fibroblast type for each target. Green arrowheads indicate the representative cells of PDGFRα^+^/DPP4^+^ around the mammary gland ducts going through the connective tissue region. Yellow arrowheads indicate the representative cells among the adipose tissue for each target. Scale bar = 500 μm (upper) and 100 μm (lower).

The characterization of the single-cell clusters recognized *Dpp4* as a highly specific marker for the Fib_0 cells (Figure 2B), whereas all populations expressed *Pdgfra*. Additionally, the Fib_0 cells showed higher expression of *Dpp4* than any of the other types of cells in the mammary glands (e.g., *Epcam* ^+^ epithelial cells and *Ptprc* ^*+*^ immune cells) (Supplementary Figure S2D). Therefore, to identify the localization of the two major types of mammary gland fibroblasts, *Dpp4* ^*+*^ and *Dpp4* ^*-*^ fibroblasts, defined from our scRNA-seq analysis, we performed immunostaining in two adjacent sections of normal mouse mammary gland tissue for DPP4 as the Fib_0-specific marker, and PDGFRα as the pan-fibroblast marker (Figure 3). From the hematoxylin and eosin (H&E) staining and immunostaining for PDGFRα (Figures 3A,B), we found that the PDGFRα^+^ fibroblasts were mainly located in two regions: in the connective tissues around and/or within the fat pad (Figure 3A, red) and a region adjacent to the mammary gland ducts within the fat pad (Figure 3A, green). In the immunohistochemical staining, we observed that the fibroblasts in the connective tissue co-expressed PDGFRα and DPP4, while most of the fibroblasts around the ducts within the fat pad were positive only for PDGFRα (Figures 3B,C, red arrowhead). Notably, some mammary gland ducts extended through the connective tissue within the fat pads, and the fibroblasts around these ducts were also positive for both DPP4 and PDGFRα (Figures 3B,C, green arrowhead), suggesting that all the fibroblasts in the connective tissues, around and/or within the fat pad, shared characteristics with *Dpp4* ^*+*^ fibroblasts regardless of contact to any mammary ducts. Also, we found some PDGFRα^+^/DPP4^-^ cells among the mature adipocytes (Figures 3B,C, yellow arrowhead), which might be preadipocytes or immune cells (e.g., macrophages) existing in the fat pad, and further investigation would be required to exactly elucidate what kind of cells they were. In summary, our results from scRNA-seq and the following histological evaluation indicated that there were two major types of mouse mammary gland fibroblasts, *Dpp4* ^*+*^ and *Dpp4* ^*-*^ fibroblasts, with distinct functional characteristics for mammary gland organization as well as discrete localization within the mouse mammary gland.

### E2 Treatment Affected Gene Expression Profiles of Mouse *Dpp4* ^*+*^ Fibroblasts and a Subcluster of *Dpp4* ^*-*^ Fibroblasts in an Indirect and Population-Specific Manner

**FIGURE 4:**
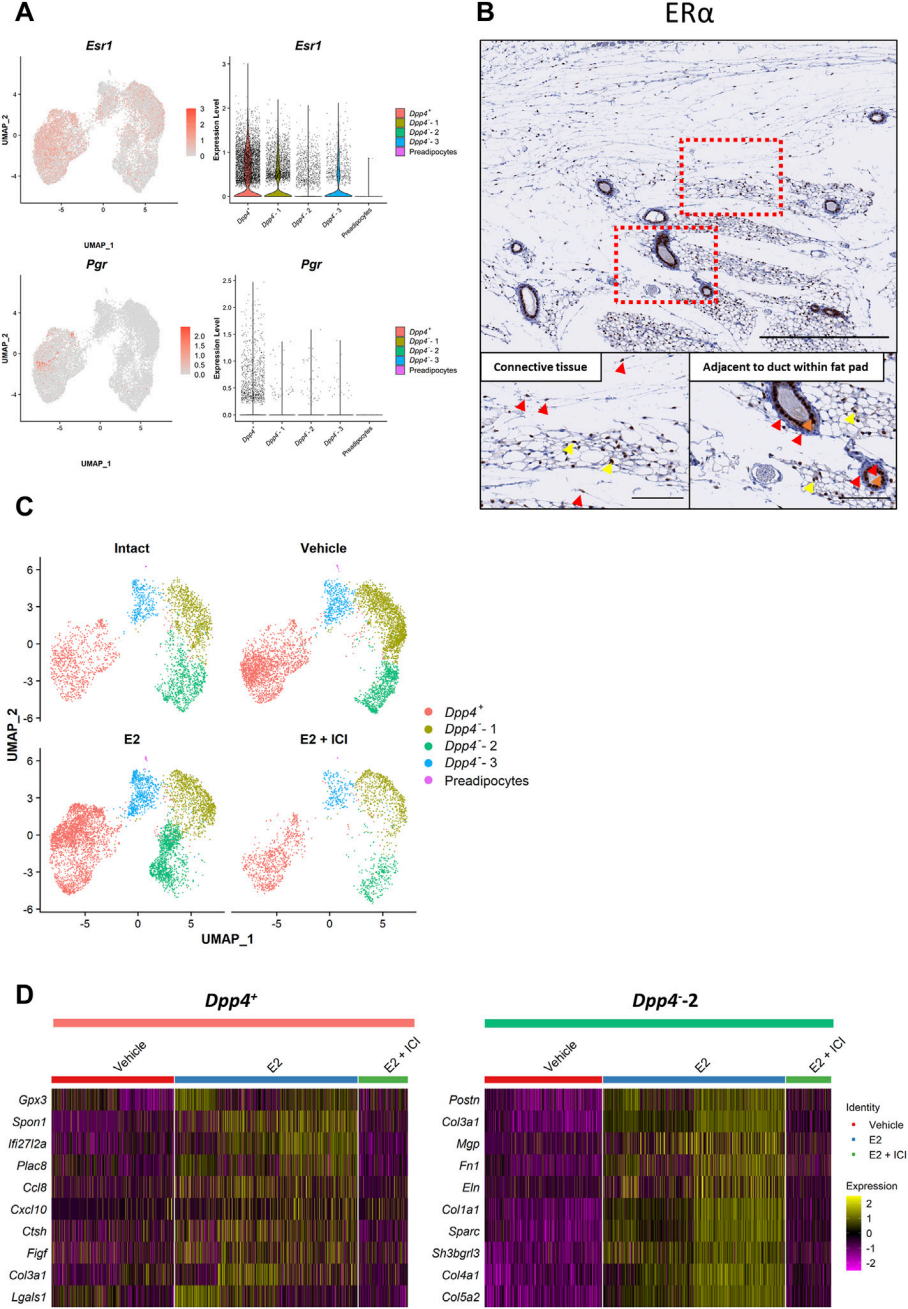
Estrogen treatment affects the gene expression profiles of the *Dpp4* ^*+*^ and *Dpp4* ^-^-2 fibroblasts in an indirect and population-specific manner. **(A)** The expression of the ovarian hormone receptors (*Esr1* and *Pgr*). *Esr2* expression was not detected in the dataset. Each feature plot on the left panel shows the distribution of the marker-expressing cells with color indicating the expression level. Each violin plot on the right panel shows the expression level of each gene in each cluster. **(B)** The immunohistochemical staining for ERα in the normal mouse mammary gland. The mammary glands of eight-week-old C57BL/6J mice were collected together with skin (at the top part of the images) and cross-sectioned. The upper panels show the entire images of the mammary glands at low magnifications. The lower panels show the magnified images of the boxed area for the connective tissue region around/within the mammary fat pad (left) and the region adjacent to the mammary gland ducts within the fat pad (right). Red arrowheads indicate the representative staining pattern of each fibroblast type. Yellow arrowheads indicate the positive staining in the cells among the adipose tissue. Orange arrowheads indicate the positive staining in the mammary luminal epithelial cells. Scale bar = 500 μm (upper) and 100 μm (lower). **(C)** The UMAP plot for the cells from each treatment group. “Intact” (top left) and “E2 + ICI” (bottom right) includes the cells from the intact (Intact) and E2+ICI group (VCD_E2_ICI) of the VCD model, respectively. “Vehicle” (top right) includes the cells from the vehicle groups of the OVX and VCD models (OVX_vehicle and VCD_vehicle). “E2” (bottom left) includes the cells from the E2 and E2+P4 groups of the OVX and VCD models (OVX_E2, OVX_E2_P4, VCD_E2 and VCD_E2_P4). Each cell is represented by each dot and is colored according to the clusters. **(D)** The heatmap of the top 10 most-upregulated genes in the E2-treated cells compared to the vehicle-treated cells in the *Dpp4* ^*+*^ and *Dpp4* ^-^-2 fibroblasts. Each column represents each cell and each row represents each gene. Log-normalized gene count was scaled per gene. Color bar at the top indicates the cell types (*Dpp4* ^*+*^ fibroblasts, pink; *Dpp4* ^-^-2 fibroblasts, green) and treatment groups (vehicle, red; E2, blue; E2 + ICI, green).

To logically examine the response of mouse mammary gland fibroblasts to ovarian hormone treatments, we first checked the hormone receptor gene expressions (Figure 4A). *Esr1*, which encodes estrogen receptor α (ERα), was expressed in both types of fibroblasts, but was more significant in the *Dpp4* ^*+*^ fibroblasts (Fib_0) compared to the *Dpp4* ^*-*^ fibroblasts (Fib_1–3). No cells expressed *Esr2* (encoding for ERβ), and very few cells expressed *Pgr* (encoding for progesterone receptor). No attempts were made to further analyze this very small number of *Pgr* ^*+*^ cells. To validate ERα expression at the protein level, we performed immunostaining of ERα on mouse mammary gland tissue (Figure 4B). We observed that some of the fibroblasts in the connective tissue expressed ERα, whereas most of the fibroblasts adjacent to the ducts were negative for ERα (Figure 4B, red arrowhead). Again, ERα staining was also observed the cells within the fat pad, where ERα^+^ preadipocytes and immune cells would be located (Figure 4B, yellow arrowhead). The intense staining of the luminal cells of the mammary ducts (Figure 4B, orange arrowhead) validated the consistency of the current results to our previous findings (Kanaya et al., 2019). These results from immunostaining analyses supported the observation in our scRNA-seq results that there were more ERα^+^ fibroblasts in the *Dpp4* ^*+*^ cluster than in the other cluster (i.e., *Dpp4* ^*-*^ fibroblasts) in mouse mammary gland stroma.

In our scRNA-seq analysis, E2 treatment in both models (OVX_E2 and VCD_E2) predominantly affected the distribution of fibroblasts on the UMAP plot, especially in the *Dpp4* ^*+*^ fibroblasts and the Fib_2 subcluster of the *Dpp4* ^*-*^ fibroblasts (*Dpp4* ^-^-2 fibroblasts) (Supplementary Figure S3A). However, the E2 + P4 treatment (OVX_E2_P4 and VCD_E2_P4) did not make remarkable changes in the fibroblast distribution when compared to E2 treatment alone. These results indicated that the addition of P4 to E2 treatment did not have much of an influence on the gene expression profiles of the mammary gland fibroblasts. Moreover, when we analyzed the DEGs between the E2 + P4 group and the vehicle group in each model (Supplementary Figures S3B, S3C), more significantly affected genes, which were located at the upper right or left edge of the volcano plots, were consistently regulated in the E2 group. From these observations, we considered that the effects of P4 on mouse mammary gland fibroblasts in the presence of E2 were less prominent. Therefore, to define the effect of E2 on gene expressions of each population of mammary gland fibroblasts, we combined the data from each model into “Intact” (intact), “Vehicle” (OVX_vehicle and VCD_vehicle), “E2” (OVX_E2, OVX_E2_P4, VCD_E2, and VCD_E2_P4), and “E2 + ICI” (VCD_E2_ICI) groups (Supplementary Figure S3A) and then performed DEG analysis and ssGSEA scoring. In the combined plot, E2 treatment changed the distribution of the *Dpp4* ^*+*^ fibroblast cluster and the *Dpp4* ^-^-2 fibroblasts (Figure 4C). However, the changes were not apparent in the *Dpp4* ^-^-1 and -3 clusters, indicating that E2 treatment did not significantly change the gene expression in these populations. Strikingly, ICI treatment reversed the distribution of cells as depicted in the vehicle group, demonstrating that the changes in gene expressions associated with E2 treatment occurred through E2-ER signaling pathway. Also, the fibroblasts from the intact group, which were exposed to physiological levels of E2, were shown to be evenly distributed within each cluster. To further elucidate the effect of E2 on these two populations of mammary gland fibroblasts, we compared the gene expression profiles and ssGSEA scores between the vehicle and E2 group cells within the *Dpp4* ^*+*^ fibroblasts and the *Dpp4* ^-^-2 fibroblasts (Figure 4D and Table 2). In the *Dpp4* ^*+*^ fibroblasts, E2 treatment induced interferon (IFN)-regulated genes, such as *Ifi27l2a* and *Cxcl10*, as well as immune-modulatory or angiogenic factors (e.g., *Ccl8*, *Figf*, and *Lgals*) as shown in the top 10 upregulated-gene list (Figure 4D). Correspondingly, in the ssGSEA, the *Dpp4* ^*+*^ fibroblasts upregulated INTERFERON_ΑLPHA_RESPONSE and INTERFERON_GAMMA_RESPONSE gene sets upon E2 treatment (Table 2). On the other hand, *Dpp4* ^-^-2 fibroblasts with E2 increased the expression of various collagen genes (*Col1a1*, *Col3a1*, *Col4a1*, and *Col5a2*) and other ECM genes (*Postn*, *Mgp*, *Fn1*, *Eln*, and *Sparc*) (Figure 4D). Also, the *Dpp4* ^-^-2 fibroblasts in the E2 group upregulated the gene signature related to ECM production (EPITHELIAL_MESENCHMAL_TRANSITION) (Table 2). However, in both the *Dpp4* ^*+*^ and *Dpp4* ^-^-2 fibroblasts, E2 treatment did not have much of an impact on the expression of estrogen-regulated gene signatures (i.e., ESTROGEN_RESPONSE_EARLY and _LATE). These results suggested that E2 showed population-specific effects on both the *Dpp4* ^*+*^ and *Dpp4* ^-^-2 fibroblasts, but the changes of the gene expressions were induced possibly through an indirect or non-classical manner, even in the ERα-expressing *Dpp4* ^*+*^ fibroblasts.

**TABLE 2:** Top 5 significant gene signatures and mean ssGSEA scores upregulated by E2 treatment.

Pathway	Mean (Vehicle group)	Mean (E2 group)	Adjusted *p* value
Upregulated by E2 in *Dpp4* ^*+*^ fibroblasts			
INTERFERON_ALPHA_RESPONSE	0.278	0.368	3.8E-189
EPITHELIAL_MESENCHYMAL_TRANSITION	0.433	0.478	3.2E-152
INTERFERON_GAMMA_RESPONSE	0.294	0.352	3.3E-147
COMPLEMENT	0.272	0.302	3.7E-141
APOPTOSIS	0.364	0.399	4.9E-131
Upregulated by E2 in *Dpp4* ^-^-2 fibroblasts			
OXIDATIVE_PHOSPHORYLATION	0.401	0.538	1.3E-183
PROTEIN_SECRETION	0.381	0.485	2.6E-171
EPITHELIAL_MESENCHYMAL_TRANSITION	0.449	0.527	8.8E-156
DNA_REPAIR	0.249	0.326	1.5E-155
ANGIOGENESIS	0.378	0.455	2.5E-153

### Integrative Analyses Combined With Mouse Fibroblast Atlas Revealed the Uniqueness of the Differentiation Processes Among Mammary Gland Fibroblasts

**FIGURE 5:**
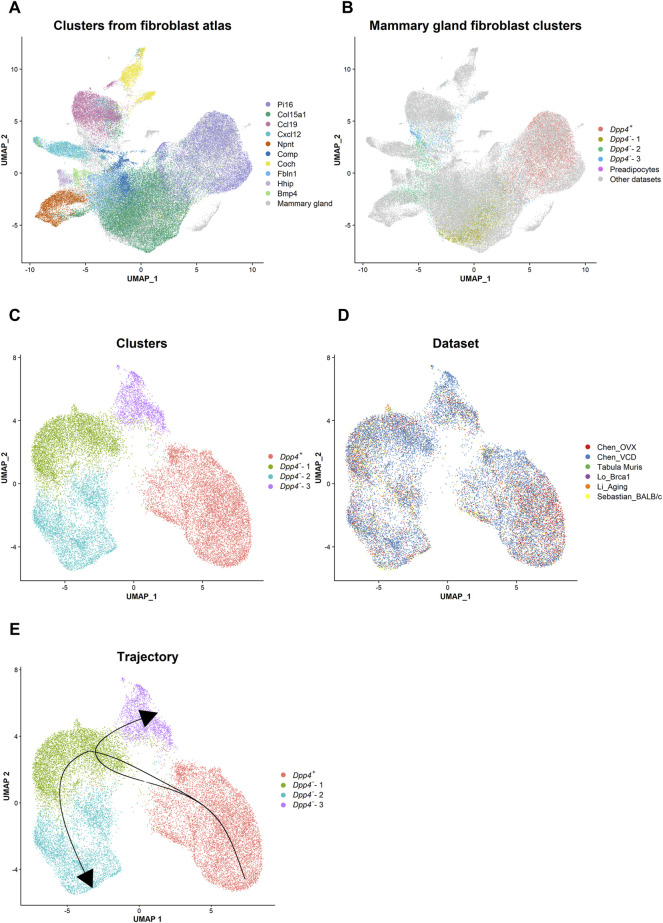
Integrative analysis reveals the lineage trajectory of mouse mammary gland fibroblasts. **(A)** The distribution of the steady state fibroblast clusters (Buechler et al., 2021) in the UMAP plot after the data integration with the mammary gland fibroblast datasets using the Harmony R package. Each cell is represented by each dot (*n* = 82,410) and is colored according to the clusters identified in the paper from Buechler et al., 2021. Mammary gland fibroblasts are colored in gray. **(B)** The distribution of the mammary gland fibroblast clusters identified in our own dataset in the UMAP plot. Each cell from our dataset (Chen_OVX/VCD) is represented by each dot and is colored according to the mammary gland fibroblast clusters as indicated in Figure 1B. The cells from the other datasets are colored in gray. **(C,D)** The UMAP plot of the integrated mammary gland fibroblast dataset. Each cell is represented by each dot (*n* = 21,861) and is colored according to **(C)** the cluster identified by the unbiased clustering and **(D)** the originated datasets. **(E)** The lineage trajectory of mammary gland fibroblasts inferred by the Slingshot R package. The principal curve was generated based on the pseudotime shown in Supplementary Figure S4C.

A recent study generated a “fibroblast atlas” by integrating the fibroblast scRNA-seq data from various mouse organs in steady states (Buechler et al., 2021). In this mouse fibroblast atlas, there are “universal” fibroblasts and “specialized” fibroblasts. The universal fibroblasts, or *Pi16* ^*+*^ (also *Dpp4* ^*+*^) and *Col15a1* ^*+*^ fibroblasts, exist across the tissues and are suggested to serve as the progenitor for the specialized fibroblasts. The reported specialized fibroblasts (e.g., *Ccl19* ^*+*^, *Npnt* ^*+*^, or *Fbln* ^*+*^) can be found in a tissue-specific manner and present selective gene expressions (Buechler et al., 2021). Since the atlas dataset by Buechler et al. (2021) did not include mammary gland fibroblasts, we integrated their steady-state mouse fibroblast atlas dataset with our own, as well as four other scRNA-seq datasets containing normal mouse mammary gland fibroblasts (Schaum et al., 2018; Li et al., 2020; Lo et al., 2020; Sebastian et al., 2020) (Figures 5A,B). After completing data integration using the Harmony R package (Korsunsky et al., 2019), we confirmed that the cluster distribution from the published atlas was well maintained on the UMAP plot (Figure 5A). When we visualized the distribution of the mammary gland fibroblast populations among the clusters from the fibroblast atlas (Figure 5B), our *Dpp4* ^*+*^ and *Dpp4* ^-^-1 fibroblasts were included in the *Pi16* ^*+*^ and *Col15a1* ^*+*^ universal fibroblast clusters, respectively. On the other hand, the *Dpp4* ^-^-2 and -3 fibroblasts from our dataset were distributed among the specialized fibroblast clusters from other organs/tissues in the fibroblast atlas. Intriguingly, these *Dpp4* ^-^-2 and -3 cells were located among the reported different fibroblast clusters, indicating that the fibroblasts in mammary glands would include separated lineages of specialized fibroblasts.

Then, to infer the differentiation trajectory within mouse mammary gland fibroblasts, we re-integrated only the mammary gland fibroblast datasets (ours and those by Schaum et al., 2018; Li et al., 2020; Lo et al., 2020; Sebastian et al., 2020; Saeki et al., 2021) (Figures 5C–E). After the integration, we identified four clusters (Figure 5C). Importantly, even after including the other datasets, the results presented a very similar clustering and marker expression pattern as compared to the results from our own datasets (Chen_OVX/VCD) (Supplementary Figures S4A, S4B). Therefore, we kept the same definitions as the populations determined from the analyses on our own datasets (i.e., *Dpp4* ^*+*^ and *Dpp4* ^-^-1 to -3) for these newly integrated clusters. The cells from individual dataset were evenly distributed in each cluster (Figure 5D), demonstrating that the findings from our analysis were consistently observed in mouse mammary glands. Using this integrated mammary gland fibroblast dataset, we performed a trajectory and pseudotime inference analysis using the Slingshot R package (Street et al., 2018). Considering the similarity of the *Dpp4* ^*+*^ fibroblasts to the universal fibroblast and the *Dpp4* ^-^-3 fibroblasts to one of the specialized fibroblasts in the fibroblast atlas (Figure 5B), we set the *Dpp4* ^*+*^ population as a starting point and *Dpp4* ^-^-3 population as an ending point. The results showed a lineage trajectory that went from the *Dpp4* ^*+*^ population to the *Dpp4* ^*-*^-1 population and then branched toward either the *Dpp4* ^-^-2 or the *Dpp4* ^-^-3 populations (Figure 5E and Supplementary Figure S4C). Collectively, these results indicated that the *Dpp4* ^*+*^ fibroblasts would serve as a progenitor population in mouse mammary glands that then differentiate into more specialized the *Dpp4* ^-^-2 and -3 fibroblasts through an intermediate state of the *Dpp4* ^-^-1 fibroblasts.

### Cell-Cell Interaction Inference Using Our Comprehensive Datasets Predicted Potential Ligand-Receptor Pairs Essential for Epithelial-Fibroblast Interaction

**FIGURE 6:**
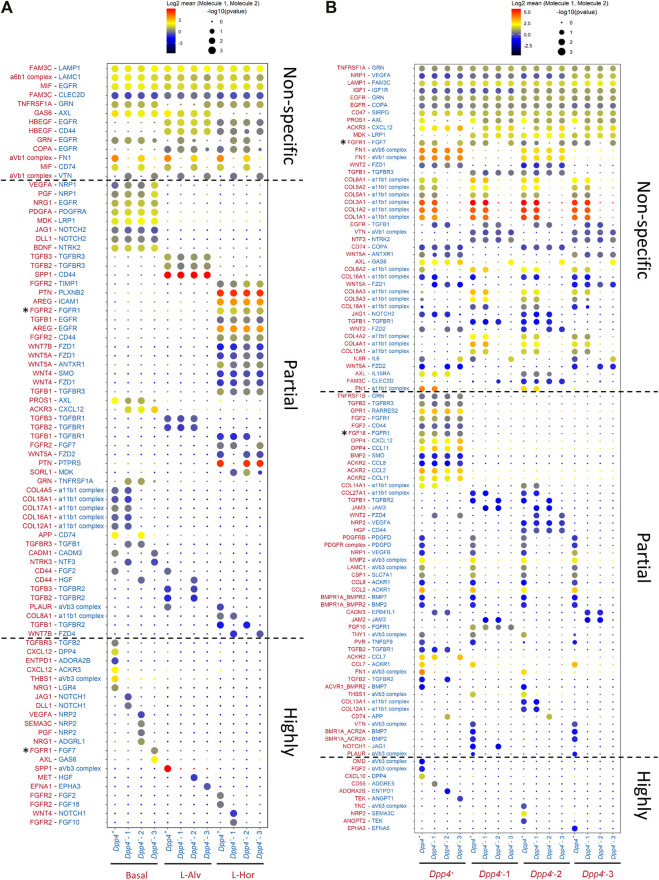
Cell-cell interaction inference. **(A,B)** The significant ligand-receptor pairs between **(A)** mammary epithelial cells and fibroblasts and **(B)** fibroblasts and fibroblasts. Size and color of each dot represent the *p* value and the level of log2 mean expression of the identified ligand-receptor pair in each row between the cell types in each column, respectively. The color of the molecules in row is matched with the cell type expressing the molecules indicated in column. The asterisks (*) represent the FGFR/FGF-related interaction pairs which were identified by the cell-cell interaction inference but were considered to be biologically improbable interactions based on the previous publications (Bellot et al., 1991; Ornitz et al., 1996; Zhang et al., 2006).

Mammary gland fibroblasts closely interact with epithelial cells to maintain or develop the mammary gland. Therefore, we performed a cell-cell interaction inference between mouse mammary fibroblasts and epithelial cells using CellPhoneDB (Efremova et al., 2020). The CellPhoneDB is a repository which curates ligands, receptors, and their mutual interactions based on publicly available databases (e.g., UniProt, Ensembl), as well as through literature mining by the developers, to infer possible cell-cell communications among given cellular populations based on the ligand-receptor expression profiles. For this analysis, we used the integrated mouse mammary fibroblast dataset generated in Figure 5C and a mouse mammary epithelial cell dataset reported in our previous paper (Saeki et al., 2021). This comprehensive epithelial dataset includes mouse epithelial cells from three lineages [i.e., basal, luminal alveolar (L-Alv), and luminal hormone-sensing (L-Hor)] (Supplementary Figure S5A). These three epithelial lineages originate from embryonic mammary stem cells, derived from unipotent progenitors in postnatal glands. Each population expresses marker genes as previously reported, such as *Krt14* in the basal cells, *Krt18*/*Csn3* in the L-Alv cells, and *Krt18*/*Esr1/Pgr* in the L-Hor cells (Supplementary Figure S5B). The analysis identified potential ligand-receptor pairs between the epithelial cells and the fibroblasts, as well as among the fibroblasts. The number of the pairs identified by this analysis was higher between the fibroblasts themselves, followed by between the fibroblasts and the epithelial cells (Supplementary Figure S5C). We found that there were three types of interaction pairs according to their specificities (Figures 6A,B). At the top of the list, there were “non-specific” pairs which could be observed among almost all epithelial and fibroblast populations. In the middle section, we identified “partially specific” pairs which were specific for the epithelial subtypes, but not for the fibroblast subtypes, or vice versa. Lastly, “highly specific” pairs at the bottom indicated that these were found only in certain combinations of the epithelial and fibroblast subtypes. Most of the partially specific pairs between the epithelium and fibroblasts were more selective for epithelial cell types, but not for fibroblast types (Figure 6A). These epithelial subtype-specific molecules included growth factors (e.g., *Nrg1*, *Pdgfa*, and *Mdk*), Notch ligands (e.g., *Dll1* and *Jag1*), and collagens in the basal cells, *Spp1* and TGFβ family genes in the L-Alv cells, and *Areg*, *Ptn*, and Wnt ligands (e.g., *Wnt4*) in the L-Hor cells. Of note, among these ligand-receptor pairs, we could identify a known epithelial-fibroblast interaction, amphiregulin (AREG)-epidermal growth factor receptor (EGFR), between the L-Hor cells and fibroblasts (Supplementary Figure S6A). This AREG-EGFR interaction was previously demonstrated to be important for the organization of mammary gland and the expression of AREG is upregulated by estrogen (Ciarloni et al., 2007). Highly specific pairs pointed to the possible cell-type-specific communication between the mammary epithelium and fibroblasts. Intriguingly, CXCL12-DPP4 was identified as a significant interaction pair between the basal cells and the *Dpp4* ^*+*^ fibroblasts (Supplementary Figure S6B), suggesting the functional importance of *Dpp4* expression in this type of fibroblasts toward the basal cells. Among the ligand-receptor pairs with L-Alv cells, the results suggested that there was hepatocyte growth factor (HGF) receptor (MET)-HGF expression between the L-Alv cells and the *Dpp4* ^*-*^-2 fibroblasts (Supplementary Figure S6C). Importantly, HGF was upregulated by E2 treatment in the *Dpp4* ^-^-2 fibroblasts (Supplementary Figure S6D). Also, several cell-type-specific interactions through fibroblast growth factor (FGF)-FGF receptor (FGFR) were identified between the L-Hor cells and fibroblasts. Among the FGF genes, *Fgf2/18* was selectively expressed in the *Dpp4* ^*+*^ fibroblasts and *Fgf10* was in the *Dpp4* ^-^-1 fibroblasts (Supplementary Figure S6E). In the epithelial cells, *Fgfr2* was specifically expressed in the L-Hor cells, although *Fgfr1* (the other subtype of FGFR gene) was expressed in all epithelial cells. Additionally, *Fgf10* was elevated in the E2-treated *Dpp4* ^-^-2 fibroblasts (Supplementary Figure S6F), although it was not listed on Figure 6A. Furthermore, the significant pairs between the fibroblast subtypes included many ECM-integrin complex (e.g., collagens and α11β1 complex), FGFR1-FGFs, chemokine pathways (e.g., *Ccl2*, *Ccl7*, *Ccl11*, *Cxcl12*), and TGFβ signaling (Figure 6B). Again, FGF genes were expressed in a population-specific manner, while *Fgfr1* expression was observed in all fibroblast populations (Supplementary Figures S6E, S6G). Among the chemokines identified, we previously reported that *Ccl2* was a fibroblast-derived and estrogen-induced factor which led to macrophage recruitment (Kanaya et al., 2019), and we found it to be upregulated in the E2-treated *Dpp4* ^*+*^ fibroblasts in our current study as well (Supplementary Figure S6H), suggesting that *Ccl2* might also be important in regard to fibroblast-fibroblast interactions, especially in the presence of estrogen. Although there was few direct evidence about the interactions among fibroblasts in mouse mammary gland, our analysis would indicate potential communication within themselves. As a note, the results included some FGFR/FGF-related interaction pairs which might be biologically improbable (Figure 6, asterisks). For example, FGFR1-FGF7 between the basal cells and the *Dpp4* ^-^-3 fibroblasts or FGF18-FGFR1 among the fibroblasts would not occur in biological settings because previous reports using *in vitro* culture systems demonstrated minimal activities of FGF7/18 on FGFR1 (Ornitz et al., 1996; Zhang et al., 2006). Also, although FGFR1 and FGFR2 were reported to transactivate each other (Bellot et al., 1991), this interaction would only happen within the same cell, suggesting that FGFR2-FGFR1 between the L-Hor cells and the fibroblasts was unlikely to occur. Nonetheless, our cell-cell interaction inference revealed many known, as well as unknown, ligand-receptor pairs expressed between the mammary epithelium and fibroblasts.

## Discussion

Although the roles of stromal cells, especially fibroblasts, in mammary glands have been studied in both human and mouse, the evidence about mammary fibroblast heterogeneity and their functional properties, especially the influence of estrogen, remains limited. Here, we performed a comprehensive and nonbiased scRNA-seq analysis of the mammary gland fibroblasts using our own datasets from two independent models with different mouse strains and steroid hormone interventions, as well as the four datasets from previous investigations. We examined the effects of estrogen on mammary fibroblasts using two mouse models for menopausal transition which is a window of susceptibility and with sensitive estrogen response in the mammary gland. The integrative analysis revealed two major populations of mammary fibroblasts across all datasets, defined as *Dpp4* ^*+*^ and *Dpp4* ^-^ fibroblasts, and profiled their distinct contributions to mammary gland organization. Histological evaluation indicated the distinct localization of these two types of fibroblasts within the normal mouse mammary glands. Also, we demonstrated an indirect (or non-classical) and population-specific effect of estrogen, which is an essential mediator for mammary gland development, on the fibroblasts. Recent advancement of analytical methods for scRNA-seq further allowed us to infer the differentiation trajectory of mammary fibroblast subtypes and their potential intercellular crosstalks with mammary epithelial cells.

DPP4 has been recognized as a marker of human interlobular fibroblasts that exist in the connective tissue between the TDLU structures found within human breast tissue (Atherton et al., 1992; Atherton et al., 1994; Morsing et al., 2016; Morsing et al., 2020). A study demonstrated that human DPP4^+^ interlobular fibroblasts highly produced type XIV collagen compared to the DPP4^-^ intralobular fibroblasts, thus suggesting the organizational importance of collagen fibers found within the dense fibrotic structure of the interlobular stroma (Atherton et al., 1998). Morsing et al. (2016) revealed the immune system-related signature of their DPP4^+^ interlobular fibroblasts using microarray analysis. Agreeing with these findings, our single-cell analyses revealed that mouse *Dpp4* ^*+*^ fibroblasts, with inflammatory response gene expression profiles, show similar characteristics to human interlobular fibroblasts. Since there is a technical limitation in investigating the functions of human interlobular fibroblasts in an *in vivo* setting, mouse *Dpp4* ^*+*^ fibroblasts identified in this study would serve as a valuable resource to study the biology of this fibroblast subtype in both human and mouse mammary gland stroma.

Another major type of mouse mammary gland fibroblasts was identified as *Dpp4* ^-^ cells, mainly localizing around the mammary gland ducts going through the mammary fat pad. These *Dpp4* ^-^ fibroblasts consisted of the three subclusters, *Dpp4* ^-^-1 to -3. ECM production and remodeling are the primary functions of fibroblasts in general, and the ECM remodeling is critical for the organization of mammary gland. One of the *Dpp4* ^-^ fibroblast subclusters, *Dpp4* ^-^-2, showed high expression of ECM encoding genes (e.g., *Tnc*, *Mfap4*) and the enrichment of the ECM gene signature. Furthermore, estrogen treatment increased the expression of ECM genes including many types of collagens in the *Dpp4* ^-^-2 fibroblasts, together with mammary duct expansion. Therefore, the *Dpp4* ^-^-2 fibroblasts would be essential for ECM remodeling within the mammary gland. In addition, recent studies performing scRNA-seq for the stromal compartments of subcutaneous and visceral adipose tissue have identified populations which show comparable gene expression profiles to our *Dpp4* ^-^-1 and *Dpp4* ^-^-3 fibroblasts (Hepler et al., 2018; Schwalie et al., 2018; Merrick et al., 2019). One study demonstrated that one of the populations sharing the same gene expression pattern with our *Dpp4* ^-^-1 cells possessed the highest adipogenic capacity both *in vitro* and *in vivo*, leading the authors to define this population as “adipose progenitor cells” in mouse adipose tissue (Merrick et al., 2019). Another study identified that *F3*/CD142-expressing “adipo-regulatory cells,” which shares gene expression pattern with our *Dpp4* ^-^-3 cells, were able to inhibit adipogenesis of those adipose progenitor cells (Schwalie et al., 2018). Therefore, we speculated that the *Dpp4* ^-^-1 and *Dpp4* ^-^-3 subclusters in our *Dpp4* ^-^ fibroblasts contributed to the homeostasis of the fat pad surrounding the mammary gland ducts.

Although ERα expression on mammary gland fibroblasts has been reported in previous studies (Parmar and Cunha, 2004; Mallepell et al., 2006; Feng et al., 2007), its importance during postnatal mammary gland organization has remained controversial. Earlier studies using ERα knockout mice showed that stromal ERα expression was required for mammary gland development (Mueller et al., 2002). However, later studies pointed that these earlier results were confounded by persistent activity of truncated ERα in the ERα knockout mouse model and demonstrated that epithelial ERα was essential for mammary gland morphology and stromal ERα was not required (Mallepell et al., 2006; Feng et al., 2007). Despite these efforts, the functional roles of ERα in mammary gland fibroblasts remained unclear and were not ruled out directly. In our study, we examined the effects of estrogen by comparing gene expression profiles in mammary gland fibroblasts from estrogen-deficient (OVX or VCD-treated) versus E2-exposed mice. We observed the changes in gene expression caused by E2 treatment specifically in the *Dpp4* ^*+*^ cluster and one of the *Dpp4* ^-^ subclusters (i.e., *Dpp4* ^-^-2), which could be reversed by ER antagonism. Importantly, both of our DEG and gene signature analyses revealed that these responses were not associated with the expression of typical estrogen-regulated genes, despite the ERα expression especially in the *Dpp4* ^*+*^ fibroblasts. These observations suggested that ERα in mammary gland fibroblasts would not be activated directly or be activated but lead to the transactivation of the other signaling pathways rather than the typical estrogen-regulated gene transcription. Supporting that, estrogen is suggested to affect mammary gland stromal cells through paracrine signaling by inducing AREG in mammary epithelial cells, a ligand for a receptor (EGFR) on stromal fibroblasts, leading to mammary ductal elongation (Ciarloni et al., 2007). Together, our results from nonbiased scRNA-seq analyses revealed that mammary fibroblasts are affected by estrogen in a non-classical manner or indirectly through other cells, in agreement with the previous observations.

In our analysis, estrogen treatment upregulated the expression of IFN-responsive gene signatures in the *Dpp4* ^*+*^ fibroblasts. It has been demonstrated that estrogen can activate ERα expressed on many types of immune cells to induce both IFN-α or IFN-γ productions (Kovats, 2015; Khan and Ansar Ahmed, 2016). Correspondingly, E2 treatment induced the upregulation of known IFN-regulated genes (e.g., *Ifi27l2a, Cxcl10*) in the *Dpp4* ^*+*^ fibroblasts. *Cxcl10* is a known regulator of immune microenvironment to drive migration and activation of immune cells, including macrophages (Tokunaga et al., 2018), which is also an important player for successful mammary gland morphogenesis (Rauner and Kuperwasser, 2021). Furthermore, the upregulated genes in the *Dpp4* ^*+*^ fibroblasts included immune modulatory or angiogenic factors such as *Ccl8*, *Lgals1*, and *Figf*. Thus, our results suggested that the *Dpp4* ^*+*^ fibroblasts would mainly contribute to the organization of immune microenvironment upon estrogen stimulation. On the other hand, the *Dpp4* ^-^-2 fibroblasts showed many upregulated ECM genes as well as the enrichment of an ECM-related gene signature. Among these ECM molecules, collagens are the most abundant in the mammary gland, and each of the upregulated collagens (collagen type I, III, IV, and V) has been shown to contribute to the various stages of mammary gland development (Maller et al., 2010). Although several studies have suggested that collagen synthesis and ECM reorganization in mammary gland is modulated by estrogen signaling or menstrual cycle in human and/or rodent models (Ferguson et al., 1992; Woodward et al., 2001; Hattar et al., 2009; Jallow et al., 2019), there has been no clear evidence that estrogen regulates collagen production in mammary gland fibroblasts either directly or indirectly. Therefore, our results directly indicate that mammary gland fibroblasts, especially the *Dpp4* ^-^-2 fibroblasts, regulate collagen synthesis under the presence of estrogens within mouse mammary glands. Considering the close contact between these *Dpp4* ^-^ fibroblasts and the mammary gland ducts, we speculate that the *Dpp4* ^-^-2 fibroblasts would modulate the ECM modeling around the ducts in response to estrogen and thereby help ductal elongation or branching through the mammary fat pad. Although the exact mechanisms for the gene signature changes remain to be revealed, to the best of our knowledge, this study is the first to profile the population-specific responses of mammary gland fibroblasts to estrogen in *in vivo* models. These results would provide the fundamental insights to further investigations for the importance of the indirect activation of fibroblasts during estrogen-induced ductal outgrowth.

The integrated analysis with the recently published fibroblast atlas (Buechler et al., 2021) indicated that our *Dpp4* ^*+*^ and *Dpp4* ^-^-1 fibroblasts were similar to the *Pi16* ^+^ and *Col15a1* ^+^ universal fibroblasts, respectively, which serve as the progenitor for other specialized fibroblasts. Corresponding to the lineage inference in their atlas, our trajectory and pseudotime analysis on the mammary gland fibroblast dataset showed the differentiation flow from the *Dpp4* ^*+*^ fibroblasts to the *Dpp4* ^-^-1 fibroblasts, then branching into *Dpp4* ^-^-2 and -3 fibroblasts. Intriguingly, both results suggested that mammary gland fibroblasts would include two distinct lineages of fibroblasts, which could be a distinct characteristic of the mammary gland fibroblasts against other organs. This uniqueness might be explained by that mouse mammary glands include both ductal structures and fat tissues and by how they are modulated by hormones like estrogen. Also, during the embryonic stages of mammary gland development, two types of mesenchymal tissues, a fibroblastic mesenchyme and a fat pad precursor mesenchyme, are known to be involved (Sakakura et al., 1982). Moreover, a recent paper studying mesenchymal cells in mouse adipose tissue demonstrated that their *Dpp4* ^*+*^ population, which shared similar characteristics with our *Dpp4* ^*+*^ fibroblasts, can give rise to *Fabp4* ^+^ adipogenic cells and then differentiate into both *F3* ^+^ adipo-regulatory cells and mature adipocytes. Their differentiation hierarchy from *Dpp4* ^*+*^ cells, passing through *Fabp4* ^*+*^ cells to *F3* ^*+*^ cells, would support one of our lineage trajectories among the *Dpp4* ^*+*^, *Dpp4* ^-^-1, and *Dpp4* ^-^-3 fibroblasts. Taken together, our single-cell analysis predicted the potential differentiation process between the *Dpp4* ^*+*^ and *Dpp4* ^-^ fibroblasts in mouse mammary glands and suggested that the distinction of the specialized *Dpp4* ^-^-2 and -3 fibroblasts would be necessary for the organization of mouse mammary gland structure.

As the final step for the characterization of our fibroblast populations, we analyzed ligand-receptor pairs expressed between mammary epithelial cells and fibroblasts and between fibroblasts themselves, thereby predicting the cell-cell interactions. Importantly, the results included some of the known epithelial-stromal interactions, such as AREG-EGFR between the L-Hor cells and fibroblasts. AREG is a critical mediator for mammary gland organization and is upregulated by estrogen; it binds to its receptor, EGFR, that is exclusively expressed on mammary stroma to further activate paracrine signaling back to the mammary epithelium (Ciarloni et al., 2007; Koledova et al., 2016). The MET-HGF pair between the L-Alv cells and the *Dpp4* ^-^-2 fibroblasts was another example of known epithelial-stromal interactions. HGF is recognized as a fibroblast-derived and estrogen-mediated factor (Zhang et al., 2002; Di-Cicco et al., 2015), agreeing with the results of the present study. The MET activation on mammary epithelial cells by HGF increases cell proliferation, leading to side branching during mammary gland development after birth (Garner et al., 2011; Gastaldi et al., 2013). Also, an earlier *in vitro* study suggested that HGF was required in progestin (a synthetic progesterone)-induced alveolar-like morphogenesis (Sunil et al., 2002). Our results may indicate a role of *Dpp4* ^-^-2 fibroblasts in the production of HGF toward proliferation and/or differentiation of L-Alv cells with ovarian hormones. Among the highly specific epithelial-fibroblast interaction pairs, a pair of interest was the CXCL12-DPP4 between the basal cells and the *Dpp4* ^*+*^ fibroblasts. While DPP4 is a dipeptidyl peptidase that can degrade several mediators and thereby control multiple signaling pathways (Mulvihill and Drucker, 2014), its function in the normal mammary gland is not yet totally defined. CXCL12 is known to be one of these mediators that is cleaved by DPP4 (Proost et al., 1998; Lambeir et al., 2001). In normal mouse mammary gland, CXCL12 signaling was demonstrated to promote the expansion of basal and luminal epithelium from mammary epithelial progenitor cells in the presence of progesterone (Shiah et al., 2015). Also, in human breast cancers, the CXCL12 signaling was shown to induce their malignant phenotypes (Ablett et al., 2013; Mukherjee and Zhao, 2013). Furthermore, the stroma of human breast cancer presents less DPP4 expression than normal breast tissues (Mezawa et al., 2019), and the suppression of DPP4 promotes cancer metastasis via CXCL12 signaling (Yang et al., 2019). Therefore, our results suggested the importance of *Dpp4* expression in the *Dpp4* ^*+*^ fibroblasts as functional molecules which would modulate CXCL12 produced by basal cells and regulate the epithelial differentiation or even suppress the malignant transformations of mammary epithelium.

FGF-FGFR signaling has been reported to play essential roles in mammary branching morphogenesis and epithelial differentiation (Lu et al., 2008; Parsa et al., 2008; Pond et al., 2013). Significantly, a previous study investigating the effect of FGFs in mammary gland demonstrated that FGF2 and FGF10 promote epithelial morphogenesis in distinct manners (Zhang et al., 2014). The authors discussed these subtype-specific activities of FGFs in terms of their selectivity for their cognate receptors shown in previous studies that FGF2 and FGF10 preferentially bind to FGFR1 and FGFR2, respectively (Ornitz et al., 1996; Zhang et al., 2006). Moreover, FGFR activation on mammary gland fibroblasts was reported to regulate various functions of mammary gland fibroblasts such as migration, ECM production, and paracrine signaling, supporting mammary epithelial morphogenesis (Sumbal and Koledova, 2019). Our analyses showed potential interaction between *Fgf2* and *Fgf18* on the *Dpp4* ^*+*^ fibroblasts, *Fgf10* on the *Dpp4* ^-^-1 fibroblasts, and *Fgfr2* on the L-Hor cells in cell-type-specific manners, while *Fgfr1* was expressed on all epithelial and fibroblast subtypes. Also, the *Dpp4* ^*-*^-2 fibroblasts with E2 treatment showed higher *Fgf10* expression. Although further validation considering the biological property of FGFR-FGF interactions (e.g., splice variants of FGFR with distinct activity, receptor binding specificity of FGFs) would be required, these results would provide further clues to understand the functional differences of the FGF family in mammary gland from the aspect of cell-type-specific interactions between the epithelium and fibroblasts, or among fibroblasts, as well as the influence of estrogen.

Furthermore, we found that some chemokines were included in the interactive pairs between the fibroblast subtypes. We previously reported that *Ccl2* is expressed in *Esr1* ^+^ fibroblasts and is induced by estrogen treatment, resulting in the estrogen-dependent recruitment of M2-macrophages (Kanaya et al., 2019). In this study, we also observed that E2 treatment increased the number of the *Ccl2* ^*+*^ cells in the *Dpp4* ^*+*^ population, and *Ccl2* was included in the ligand-receptor pairs between the *Dpp4* ^*+*^ fibroblasts and other types of fibroblasts. Thus, our analysis suggests the potential importance of the fibroblast-fibroblast interaction through *Ccl2* under the presence of estrogen. Although many of the chemokines found in the fibroblast-fibroblast interaction pairs are known to be involved in the crosstalk with immune cells, our results suggested their potential roles in the communication among mammary fibroblasts as well. Since there has been limited information on the cellular communications among mammary gland fibroblasts, the ligand-receptor pairs identified here will help future mechanistic studies, while also considering the localization or differentiation lineage, leading to the elucidation of substantially important fibroblast-fibroblast interactions within the mammary gland.

Although we made important findings from the scRNA-seq analysis of mouse mammary gland fibroblasts, we acknowledge that our study had several limitations. First, most of this study depended on the computational analyses of the transcriptomic data, without direct validation. However, we are pleased to see that our observations agree and support many of experimental findings reported by other investigators on mammary gland fibroblast studies (Atherton et al., 1992; Mallepell et al., 2006; Ciarloni et al., 2007; Morsing et al., 2016). In addition, our findings of the fibroblast subsets were further supported by the integrated datasets that included multiple datasets from other previously reported studies, confirming that these populations commonly existed in mouse mammary gland stroma regardless of the different settings of scRNA-seq experiments. Our study showed DPP4/PDGFRα protein expression in normal mouse mammary gland tissues, supporting the localization of the *Dpp4* ^*+*^ and *Dpp4* ^*-*^ fibroblast populations. Another point is that our cell-cell interaction inference included only mammary epithelial cells and fibroblasts, but no other major types of cells such as immune cells, endothelial cells, or adipocytes. Since there has not been any comprehensive datasets like our epithelial and fibroblast datasets, we decided to focus on these two types of cells in our present analysis. Lastly, the cell-cell interaction inference was able to only speculate the potential interactions based on the gene expression data as it included some biologically improbable ligand-receptor pairs (e.g., FGFR2-FGFR1 and FGFR1-FGF7). Therefore, further biological validation for the inferred cell-cell interactions using *in vitro* and/or *in vivo* models would strengthen the predictions from our scRNA-seq analysis. Nevertheless, our efforts represent the first attempt to evaluate epithelial-fibroblast cell interactions in the mammary gland at the single-cell level. Collectively, our study could provide better insights for the characteristics of mouse mammary gland fibroblasts despite these limitations.

**FIGURE 7:**
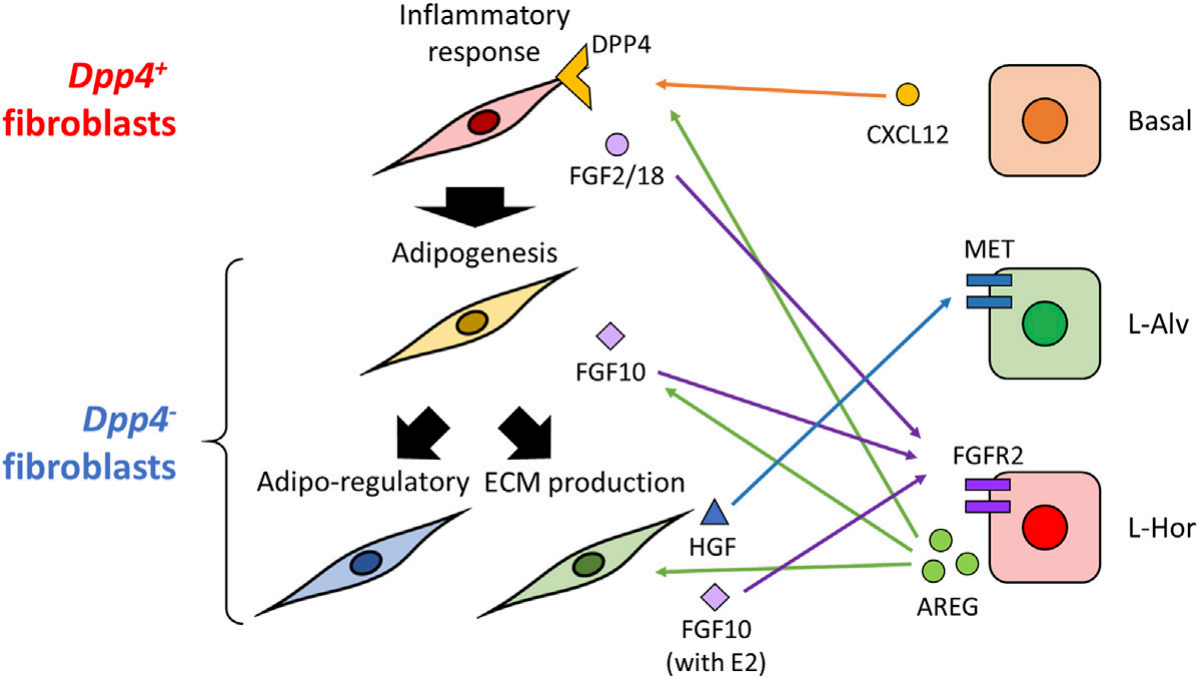
Graphical abstract of this study. In the present study, we identified two major types of mouse mammary gland fibroblasts: *Dpp4* ^*+*^ and *Dpp4* ^-^ fibroblasts. They each showed discrete gene expression profiles as indicated in this figure (e.g., inflammatory response in the *Dpp4* ^*+*^ fibroblasts), as well as distinct localization within the mouse mammary gland. Estrogen induced gene expression changes in a population-specific manner, without distinct activation of typical estrogen-regulated gene expressions. Trajectory analysis indicated a directional differentiation from the *Dpp4* ^*+*^ fibroblasts towards the subclusters of the *Dpp4* ^-^ fibroblasts as indicated by thick black arrows on the left. Cell-cell interaction inference suggested potential interactions with certain subtypes of mammary epithelial cells (e.g., AREG-EGFR, CXCL12-DPP4, MET-HGF or FGFR2-FGF2/10/18; indicated by the colored arrows).

In conclusion (Figure 7), this study firstly profiled the heterogeneity of normal fibroblasts in mouse mammary glands, which were commonly observed across various experimental settings. The identified populations, *Dpp4* ^*+*^ and *Dpp4* ^-^ fibroblasts, showed unique characteristics in gene expression profiles and localizations, suggesting their distinct contributions to mammary gland organization. Also, the analysis of our original datasets revealed the population-specific effect of estrogen on mouse mammary gland fibroblasts. The trajectory analysis, using broader and more integrative datasets such as the recently established mouse fibroblast atlas, further addressed the uniqueness in the differentiation process of mammary gland fibroblasts. Moreover, the large-scale computational inference of cell-cell interactions in combining the mammary epithelial atlas suggested potential cell-type-specific interactions (e.g., AREG-EGFR, CXCL12-DPP4, and FGF2/10/18-FGFR2) and expanded our knowledge about the potential roles of mammary fibroblasts. Our results provide fundamental insights for further investigation of the biological implications of fibroblast subsets in the mammary gland organization and eventually breast cancer pathology.

## Materials and Methods

### Animal Experiments

Female BALB/cJ and C57BL/6J mice were obtained from Jackson Laboratory (Bar-Harbor, ME). The detailed protocol for the OVX model experiment was described in our previous publication (Saeki et al., 2021). Briefly, nine-week-old BALB/cJ mice were ovariectomized, and 20 weeks after surgery, they were randomized into vehicle, E2, and E2 + P4 groups. After a week of treatment with estrogen (1 μg/animal/day) and progesterone (1 mg/animal/day) *via* intraperitoneal injection, mice were euthanized, and mammary glands were collected for following experiments. For the VCD model, nine-week-old C57BL/6J mice were treated with VCD for 2 weeks. After 34 weeks from the initial dose of VCD, mice were randomized into vehicle, E2, E2 + P4, and E2 + ICI. E2 and P4 were administered for a week as described above. For the E2 + ICI group, a single dose of ICI (5 mg/animal) was administered *via* intraperitoneal injection at the same time as the first dose of E2. Sesame oil was used as the vehicle for the VCD and hormonal treatment. After their respective treatments, mice were euthanized to collect their mammary glands. For the VCD models, mice that did not undergo the VCD treatment were included in this study as the intact group. Animal research procedures used in this study were approved by the Institutional Animal Care and Use Committee (IACUC) at City of Hope and were operated according to the institutional and National Institutes of Health (NIH) guidelines for animal care and use.

### Mammary Gland Whole-Mount Imaging

Mammary gland whole mount staining for the OVX model performed in our previous studies was re-evaluated (Saeki et al., 2021). For the VCD model, the staining was performed as described previously (Kanaya et al., 2019; Saeki et al., 2021). Briefly, mammary glands were fixed with 10% buffered formalin and defatted with toluene for 72 h. The mammary glands were rehydrated with gradient ethanol and stained with 0.025% Toluidine Blue. After being immersed sequentially in methanol, ethanol, and a 4% ammonium molybdate solution, the stained glands were dehydrated with an increasing concentration of ethanol and cleared using Histoclear (National Diagnostics, Atlanta, GA, United States) overnight. The cleared slides were mounted with Permount Mounting Medium (ThermoFisher Scientific, Waltham, MA, United States). The images of the whole mammary glands were captured using Cell3iMager Duos (SCREEN Holdings Co., Ltd., Kyoto, Japan) with 20 μm-intervals for the z-axis.

### scRNA-Seq Analysis on Our Datasets

For the OVX model, the scRNA-seq data from our previous paper (Chen_OVX) (Saeki et al., 2021) was obtained from National Center for Biotechnology Information (NCBI) Gene Expression Omnibus (GEO) data repository (GSE149949). For the VCD model, the fourth mammary glands were harvested by dissecting it from the thin muscle layer under the skin by holding the connective tissue around the gland. After lymph nodes were removed, the glands were minced with a scalpel and enzymatically digested with 1.5 mg/ml DNAse I (#10104159001, Millipore Sigma, Burlington, MA), 0.4 mg/ml Collagenase IV (CLS-4, Lot: 47E17528A, Worthington Biochemical Corporation, Lakewood, NJ, United States), 5% FBS, and 10 mM HEPES in HBSS at 37°C for an hour, while shaking at 250 rpm. After being strained through a 70 μm cell strainer, the samples were treated with ACK lysis buffer to remove residual blood cells. Dead cells were removed using Dead Cells Removal Microbeads (Miltenyl Biotec, Bergisch Gladbach, Germany). After the sample viability were ensured using TC20 Automated Cell Counter, samples with >80% viability were loaded onto the Chromium Controller (10x Genomics, Pleasanton, CA, United States) targeting 2,000–5,000 cells per lane. The Chromium v3 single-cell 3′ RNA-seq reagent kit (10x Genomics) was used to generate single-cell RNA-seq libraries according to the manufacturer’s protocol. The libraries were sequenced with the NovaSeq 6000 system (Illumina, San Diego, CA, United States) with a depth of 50 k-100 k reads per cell. Raw sequencing data were processed using the 10x Genomics Cell Ranger pipeline (version 3.1.0) and then aligned to mm10 mouse genome. The datasets generated (Chen_VCD) can be found in the NCBI GEO database under the accession GSE191219. The downstream analyses of the scRNA-seq data were performed using R scripts (version 4.0.4) and the Seurat R package (version 4.0.0), unless otherwise noted. First, the count data from low quality cells with <500 genes, < 1,000 transcripts, or >5% mitochondrial genes were excluded. Cells without *Epcam*, *Krt14*, *Ptprc*, *Cd52*, *Pecam*, and *Cspg4* gene counts were selected as described in previous studies (Bartoschek et al., 2018; Kanaya et al., 2019). To remove the batch effects between the experiments, the log-normalized count data from both datasets (Chen_OVX and Chen_VCD) were integrated using *FindIntegrationAnchors* and *IntegrateData* functions according to the developer’s vignettes. After the integrated data was scaled, a principal component analysis was performed using the top 3,000 variable genes. Based on the top principal component, UMAP dimension reduction and cluster detection with the Louvain algorithm was performed to visualize the clusters. Differentially expressed genes were identified using a logistic regression framework (*test.use =* “*LR*” in *FindAllMarkers* function) with the difference of datasets set as a latent variable (*latent.vars =* “*Dataset*”) for generating heatmaps and feature plots. ssGSEA scores for the hallmark gene sets from the MSigDB were calculated at the single-cell level using log-normalized read counts and the GSVA R package (version 1.38.2; *method =* “*ssgsea*”). For the comparison of vehicle- and E2-treated cells within the *Dpp4* ^*+*^ and *Dpp4* ^-^-2 fibroblasts, DEGs were identified using *FindConservedMarkers* function (*grouping.var =* “*Dataset*”). The comparison of ssGSEA scores either between each fibroblast population or between vehicle- and E2-treated cells within the *Dpp4* ^*+*^ and *Dpp4* ^-^-2 fibroblasts were performed by Wilcoxon rank-sum test. For the comparison of the fibroblast populations, one-side test was performed to examine the upregulated signatures in each population (*alternative =* “*greater*” in *wilcox. test* function). Multiple comparison was adjusted by Benjamini–Hochberg method.

### Histological Evaluation

The fourth mouse mammary gland of eight-weeks-old C57BL/6J mice were collected with skin and fixed with 10% buffered formalin. After embedding in paraffin, the cross-sections of the tissue were prepared and used for H&E staining and immunohistochemistry. Immunohistochemistry was performed by the Pathology Solid Tumor Core at City of Hope using Ventana Discovery Ultra IHC Auto Stainer (Roche Diagnostics, Indianapolis, IN, United States). Heat-mediated antigen retrieval was performed using Cell Conditioning Buffer 1 (Roche Diagnostics; pH 8.5) for an hour. Antibodies used for the immunostaining included: ERα rabbit polyclonal antibody (06–935, Millipore Sigma; 1:400), anti-PDGFRα rabbit monoclonal antibody (ab134123, Abcam, Cambridge, United Kingdom; 1:50), and anti-DPP4 rabbit monoclonal antibody (ab187048, Abcam; 1:500). Images were captured on the Zeiss Observer II (Carl Zeiss, Oberkochen, Germany; for H&E staining), VENTANA iScan HT (Roche Diagnostics; for PDGFRα and DPP4 immunohistochemistry) or Nano Zoomer S360 (HAMAMATSU PHOTONICS, Shizuoka, Japan; for ERα immunohistochemistry).

### Data Retrieval and Preprocessing for the Publicly Available Datasets

For the integrative scRNA-seq data analysis, a dataset including fibroblasts from various mouse organs and four datasets including mammary gland fibroblasts were obtained (Schaum et al., 2018; Li et al., 2020; Lo et al., 2020; Sebastian et al., 2020; Buechler et al., 2021). Buechler et al. (2021) integrated 28 datasets including fibroblast scRNA-seq data and established a steady-state fibroblast atlas. Their dataset was obtained from their website (https://fibroXplorer.com). A half number of cells in each cluster was randomly sampled to avoid excess memory usage during the integration process and used for the analysis. Tabula Muris is a compendium of mouse scRNA-seq data originated from 20 organs and tissues, including the mammary gland (Schaum et al., 2018). Their dataset derived from the mouse mammary gland prepared using 10x Genomics platform was retrieved from their online portal (https://tabula-muris.ds.czbiohub.org/). Fibroblast count data was extracted according to their annotation (“stromal cell”). Li et al. (2020) studied the influence of aging on mammary epithelium and stroma; their dataset was obtained from the NCBI GEO data repository (GSE150580) and their fibroblast clusters were used in this study. Lo et al. (2020) performed scRNA-seq on mammary glands from *Brca1* ^*−/−*^; *p53* ^*+/−*^ mice fed with either a regular or a high fat diet and their dataset was available on the NCBI GEO (GSE152866). Since their annotation was not available, we performed negative selection, as we did for our own datasets. Sebastian et al. (2020) analyzed both cancer-associated fibroblasts in BALB/c-derived 4T1 breast cancer and normal mammary fibroblasts in BALB/c mammary glands. The normal fibroblast dataset was available on Dryad data repository (https://datadryad.org, doi: 10.6071/M3238R). Their data was directly utilized for the data integration described in the following parts.

### Data Integration of the Fibroblast Atlas and the Mammary Fibroblast Datasets

Data integration using the fibroblast atlas datasets and the mammary gland fibroblast datasets, including ours and the others, was performed using the Harmony R package (version 0.1.0) (Korsunsky et al., 2019) according to developer’s vignette. The count data from each dataset was merged, normalized, and scaled. After the top 20 principal components were calculated from 2,000 variable features with the Seurat package, the data were integrated using *RunHarmony* function implemented in Harmony algorithm. The integrated dataset was clustered based on the calculated harmony embeddings and visualized on a UMAP plot. For the data integration using only the mammary gland fibroblast datasets, *FindIntegrationAnchors* and *IntegrateData* in the Seurat package were used. Since there was small amount of contamination from immune cells, these cells were removed and then the data integration and following cluster detection were performed again as described in the part above.

### Mammary Gland Fibroblast Trajectory Inference

Lineage trajectory and pseudotime inference was performed on the integrated mammary gland fibroblast dataset. For this purpose, we used the Slingshot R package (version 1.8.0) (Street et al., 2018). After the UMAP dimensional reduction and clustering, the integrated datasets were converted from a Seurat object to a SingleCellExperiment object (*assay =* “*RNA*”)*.* Then, lineage trajectory and pseudotime was calculated using the *slingshot* function. Considering the similarity of the *Dpp4* ^*+*^ fibroblasts to the “universal” *Pi16* ^*+*^ cluster and the *Dpp4* ^-^-3 fibroblasts to the “specialized” fibroblasts in the fibroblast atlas (Buechler et al., 2021), the *Dpp4* ^*+*^ cluster and *Dpp4* ^-^-3 cluster were set as the starting and ending clusters for the calculation, respectively. The principal curve of the inferred trajectory and the pseudotime of each cell was visualized on a UMAP plot.

### Cell-Cell Interaction Inference

Cell-cell interaction inference was performed using python (ver. 3.7.0) and the CellphoneDB python package (ver. 2.1.7) (Efremova et al., 2020). To predict the cell-cell interactions between mammary gland fibroblasts and epithelial cells, we used the integrated fibroblasts dataset and a dataset for mammary epithelium which we generated in our previous publication (Saeki et al., 2021). Ligand-receptor pairs which were significantly enriched between certain populations were calculated with the *statistical-analysis* method as implemented in developer’s Github page (https://github.com/Teichlab/cellphonedb) (Efremova et al., 2020). Threshold value for the percentage of cells expressing the ligand or receptor was set at 25% (*-threshold = 0.25*). The number of interaction pairs between the populations, log2 mean value of the gene expressions, and *p* values were visualized using the ggplot2 R package (version 3.3.3).

## Data Availability

The data presented in the study are deposited in the NCBI GEO repository (https://www.ncbi.nlm.nih.gov/geo/), accession number GSE191219.
