# Supplementary material for: Single-Cell Transcriptomics Identifies Heterogeneity of Mouse Mammary Gland Fibroblasts With Distinct Functions, Estrogen Responses, Differentiation Processes, and Crosstalks With Epithelium
Source: Front Cell Dev Biol. 2022 Mar 1;10:850568. doi: 10.3389/fcell.2022.850568 (PMC8923650; doi:10.3389/fcell.2022.850568)
Supplement: Supplementary file 1 [file DataSheet1.PDF]

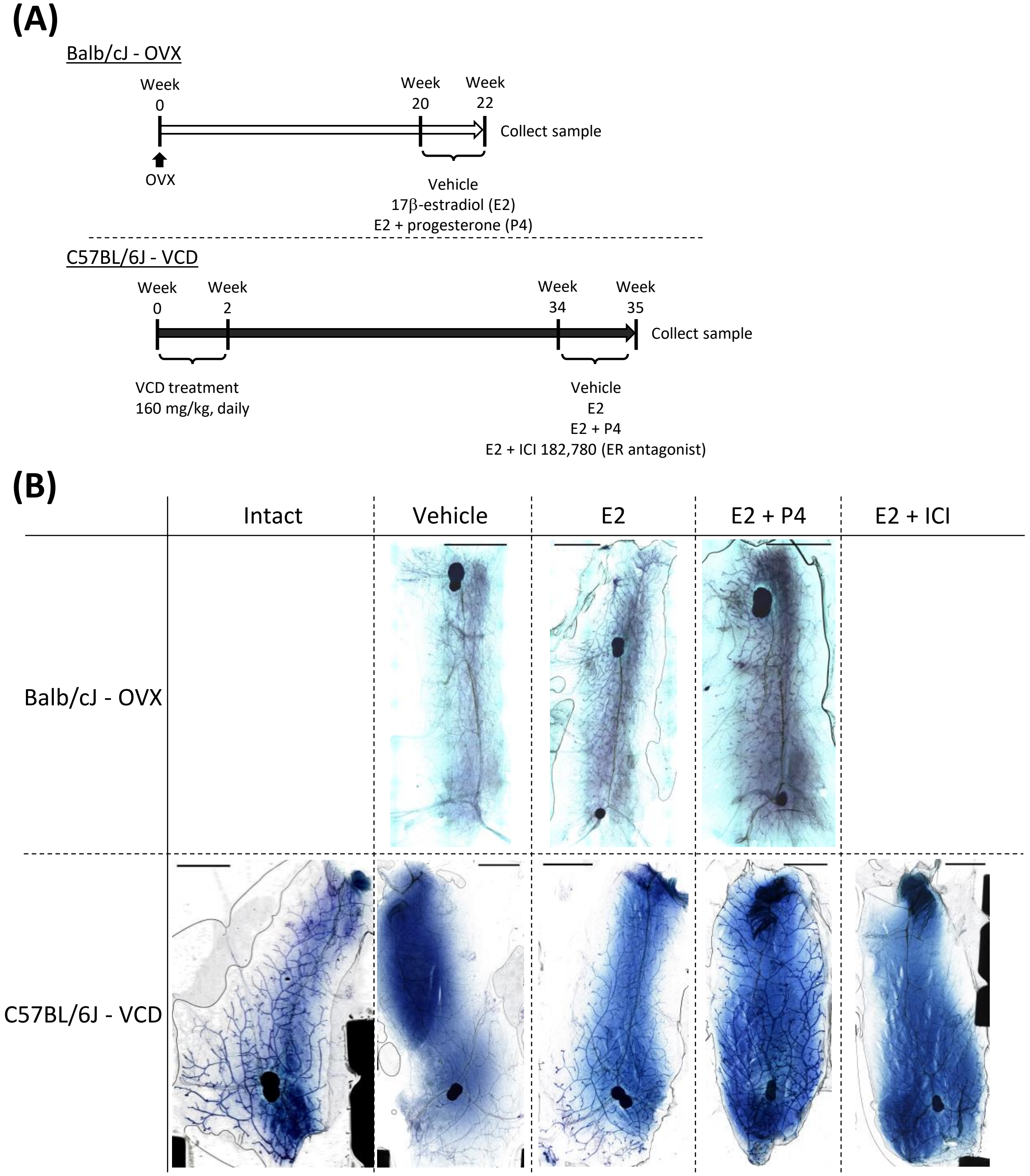

Supplementary Figure 1. Mammary gland phenotypes in the two hormone-depleted mouse models. **(A)** The overview of the experiments in the ovariectomy (OVX) and 4-vinylcyclohexene diepoxide (VCD) models. In the OVX model, female nine-week-old BALB/cJ mice were ovariectomized. 20 weeks after surgery, the mice were treated with vehicle, 17β-estradiol (E2), and E2 + progesterone (E2 + P4) for a week (Saeki et al., 2021). In the VCD model, female nine-week-old C57BL/6J mice were treated with VCD for 15 days. After 34 weeks from the onset of VCD treatment, the mice were treated with vehicle, E2, E2 + P4, and E2 + ICI 182,780 (E2 + ICI) for a week. Age-matched intact mice were also included in the VCD model. VCD (160 mg/kg), E2 (1 μg/animal), P4 (1 mg/animal) were injected daily via intraperitoneal route. A single dose of ICI (5 mg/animal) was administered via intraperitoneal route. Sesame oil was used as the vehicle for VCD and hormonal treatment. **(B)** The representative images of Toluidine blue whole mount staining of the mouse mammary glands from the vehicle, E2, and E2 + P4 groups in the OVX model, and from the intact, vehicle, E2, E2 + P4, E2 + ICI groups in the VCD model. The images for the OVX model were retrieved from our previous paper (Saeki et al., 2021). Scale bars = 5 mm.

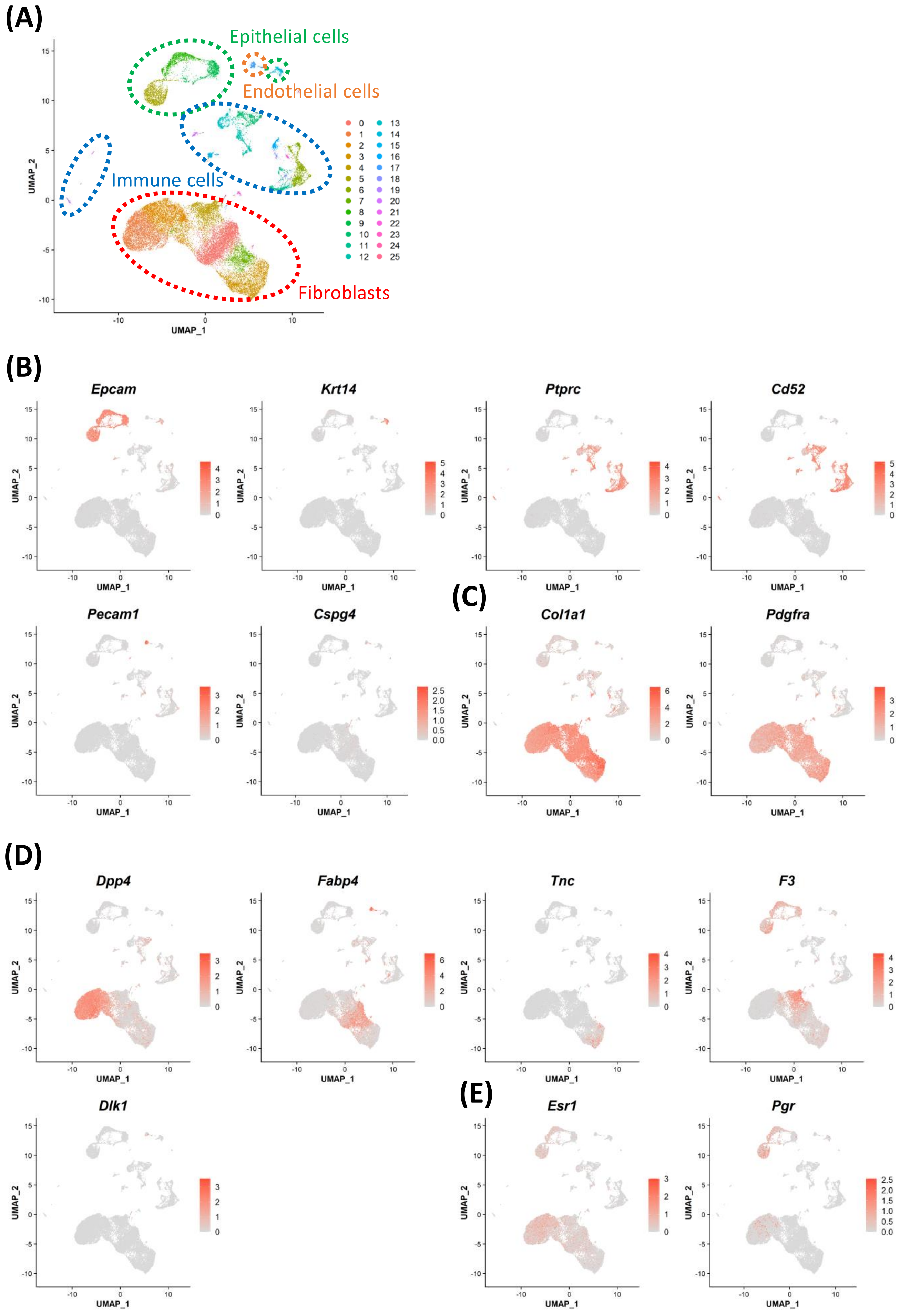
(A)
Epithelial cells
Endothelial cells
Immune cells
Fibroblasts
UMAP_1
UMAP_2
0 1 2 3 4 5 6 7 8 9 10 11 12 13 14 15 16 17 18 19 20 21 22 23 24 25
(B)
Epcam
Krt14
Ptprc
Cd52
Pecam1
Cspg4
(C)
Col1a1
Pdgfra
(D)
Dpp4
Fabp4
Tnc
F3
Dlk1
(E)
Esr1
Pgr

Supplementary Figure 2. Marker expressions in the entire dataset from the mouse mammary glands in the OVX and VCD models. **(A)** The uniform manifold approximation and projection (UMAP) plot of the entire dataset including fibroblasts and other types of cells. Each cell is represented by each dot (n = 29,452) and is colored according to the cluster identified by the unbiased clustering. The cell types indicated in dash lines were annotated according to the marker gene expression shown in **(B)**. **(B)** The expression of the marker genes for epithelial cells (*Epcam*, *Krt14*), immune cells (*Cd52*, *Ptprc*), endothelial cells (*Pecam1*), and pericytes (*Cspg4*). **(C)** The expression of the fibroblast marker genes (*Col1a1*, *Pdgfra*). **(D)** The expression of the specific markers for each fibroblast population identified in **Figure 2B** (*Dpp4*, *Fabp4*, *Mdk*, *F3*, and *Dlk1*). **(E)** The expression of the hormonal receptor genes (*Esr1*, *Pgr*; *Esr2* was not detected in this dataset). Each feature plot shows the distribution of marker-expressing cells with color indicating the expression level.

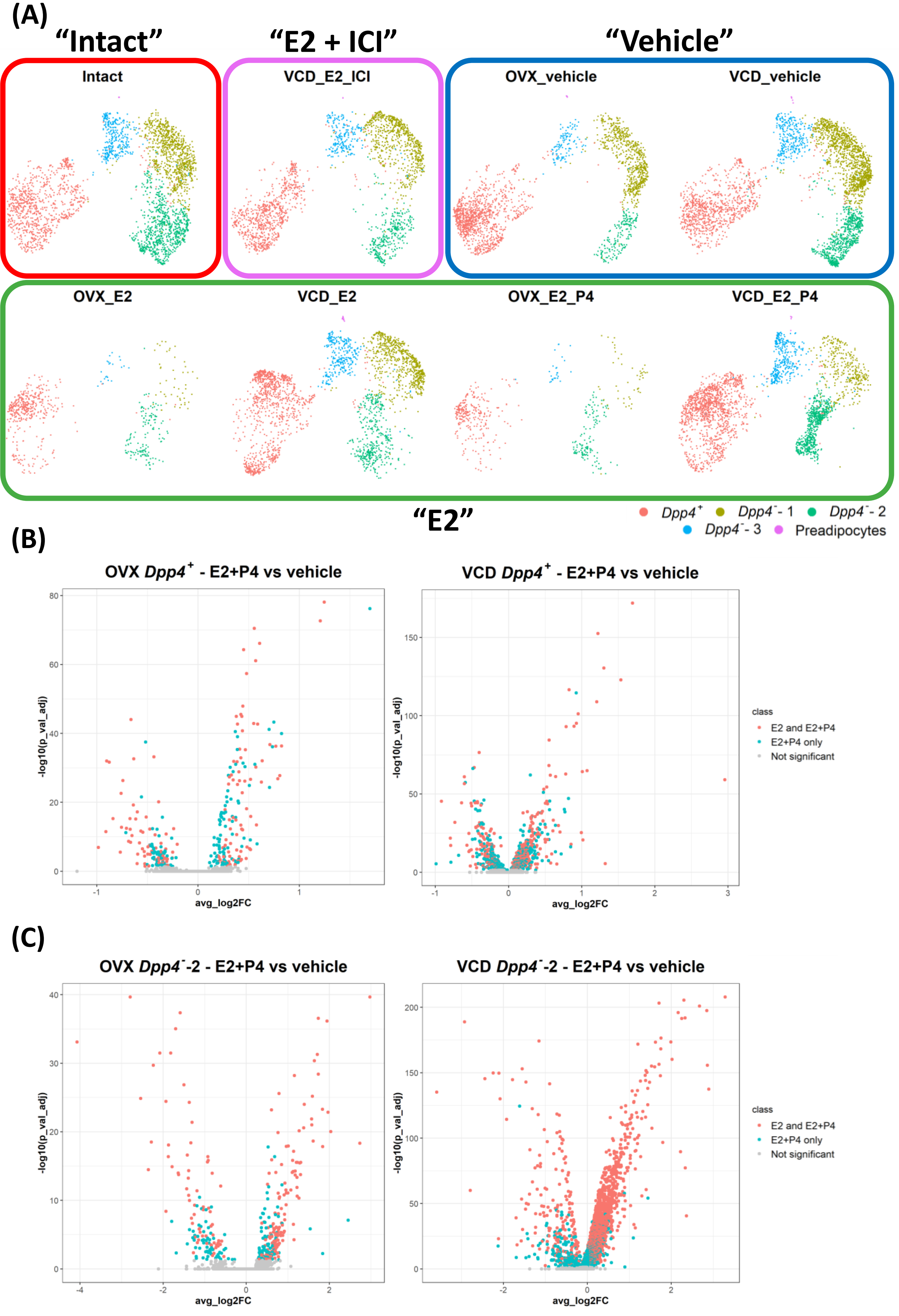

(A)
"Intact"
"E2 + ICI"
"Vehicle"
Intact
VCD_E2_ICI
OVX_vehicle
VCD_vehicle
OVX_E2
VCD_E2
OVX_E2_P4
VCD_E2_P4
"E2"
Dpp4+
Dpp4- - 1
Dpp4- - 2
Dpp4- - 3
Preadipocytes
(B)
OVX Dpp4+ - E2+P4 vs vehicle
VCD Dpp4+ - E2+P4 vs vehicle
-log10(p_val_adj)
avg_log2FC
class
E2 and E2+P4
E2+P4 only
Not significant
(C)
OVX Dpp4- -2 - E2+P4 vs vehicle
VCD Dpp4- -2 - E2+P4 vs vehicle
-log10(p_val_adj)
avg_log2FC
class
E2 and E2+P4
E2+P4 only
Not significant

Supplementary Figure 3. Difference in the cell distributions and gene expressions among the treatment groups in the OVX and VCD models. **(A)** The UMAP plot of the mammary gland fibroblasts in each treatment dataset. The cells from each group in each model are separately visualized on the same UMAP dimension (*split.by = "orig.ident"* in *UMAPPlot* function implemented in the Seurat R package). Each cell is represented by each dot and is colored according to the cluster identified in **Figure 1B**. The datasets were classified into the four groups ("Intact", "E2 + ICI", "Vehicle", and "E2") as indicated by the colored boxes and visualized in **Figure 4C**. **(B, C)** The volcano plots of the differentially expressed genes (DEGs) between the E2 + P4 groups and the vehicle groups in **(B)** the *Dpp4*$^{+}$ fibroblasts and **(C)** the *Dpp4*$^{-}$-2 fibroblasts. The left and right panels represent the results from the OVX model (OVX_E2_P4 versus OVX_vehicle) and the VCD model (VCD_E2_P4 versus VCD_vehicle), respectively. The DEGs were detected using the *FindMarkers* function (*logfc.threshold = 0*) in the Seurat R package. Each dot represents each DEG. The significant DEGs that were consistently found in the comparisons between the E2 and vehicle groups are shown in red, while the significant DEGs found only between the E2 + P4 and vehicle groups are shown in blue. Non-significant genes are shown in gray. X axis represents the average of log2 fold change value with positive values indicating higher expressions in the E2 + P4 groups compared to the vehicle groups. Y axis represents the -log10 values of adjusted *p* value. The adjusted *p* value < 0.05 was considered as significant.

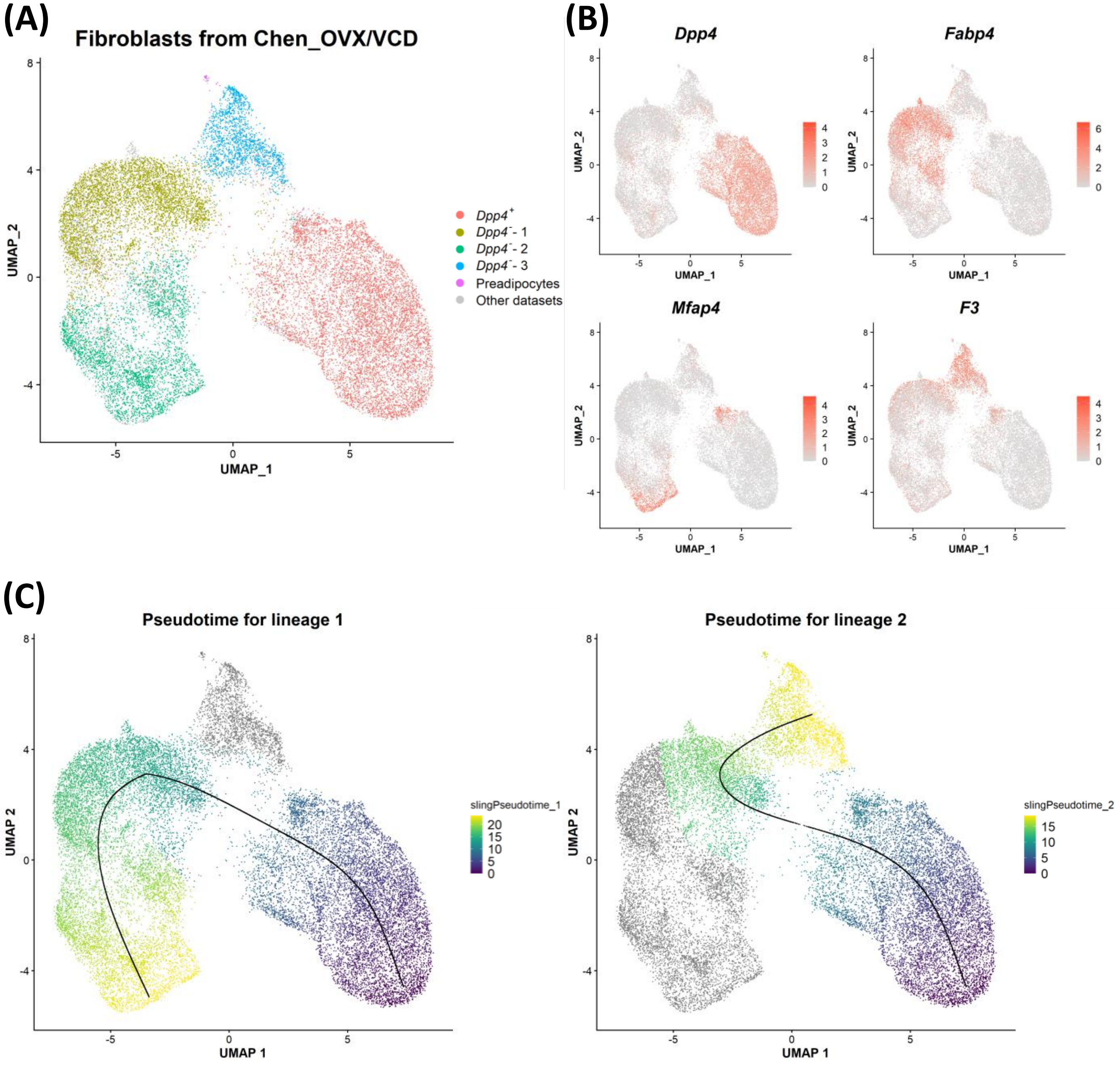

Supplementary Figure 4. Integrated analysis using our own and four other mammary gland fibroblast datasets. **(A)** The distribution of the cells from our own datasets (Chen_OVX/VCD) in the UMAP plot of the integrated mammary gland fibroblast dataset shown in **Figure 5C**. Each cell is represented by each dot and is colored according to the mammary gland fibroblast clusters as indicated in **Figure 1B**. The cells from the other datasets are colored in gray. **(B)** The expression of specific markers for each fibroblast population identified in our datasets (*Dpp4*, *Fabp4*, *Mdk*, and *F3*). Each feature plot shows the distribution of the marker-expressing cells in the integrated mammary gland fibroblast dataset. Color indicates the expression level. **(C)** The pseudotime visualization on the UMAP plot. Color indicates the pseudotime calculated by the Slingshot R package. Each curve on the UMAP plot indicates the lineage trajectory shown in **Figure 5E**.

(A)

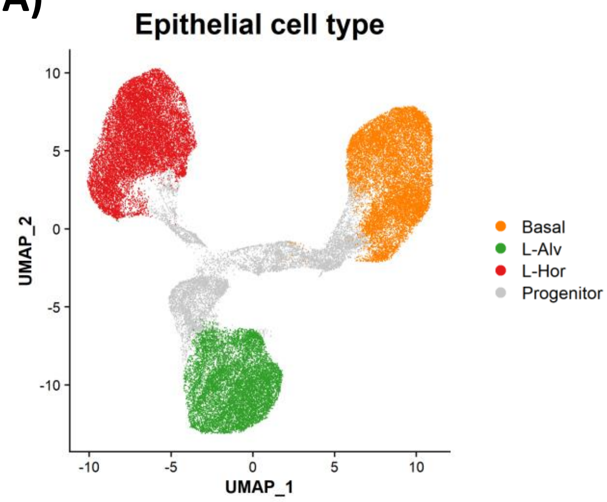

Epithelial cell type
UMAP_2
UMAP_1
Basal
L-Alv
L-Hor
Progenitor

(B)

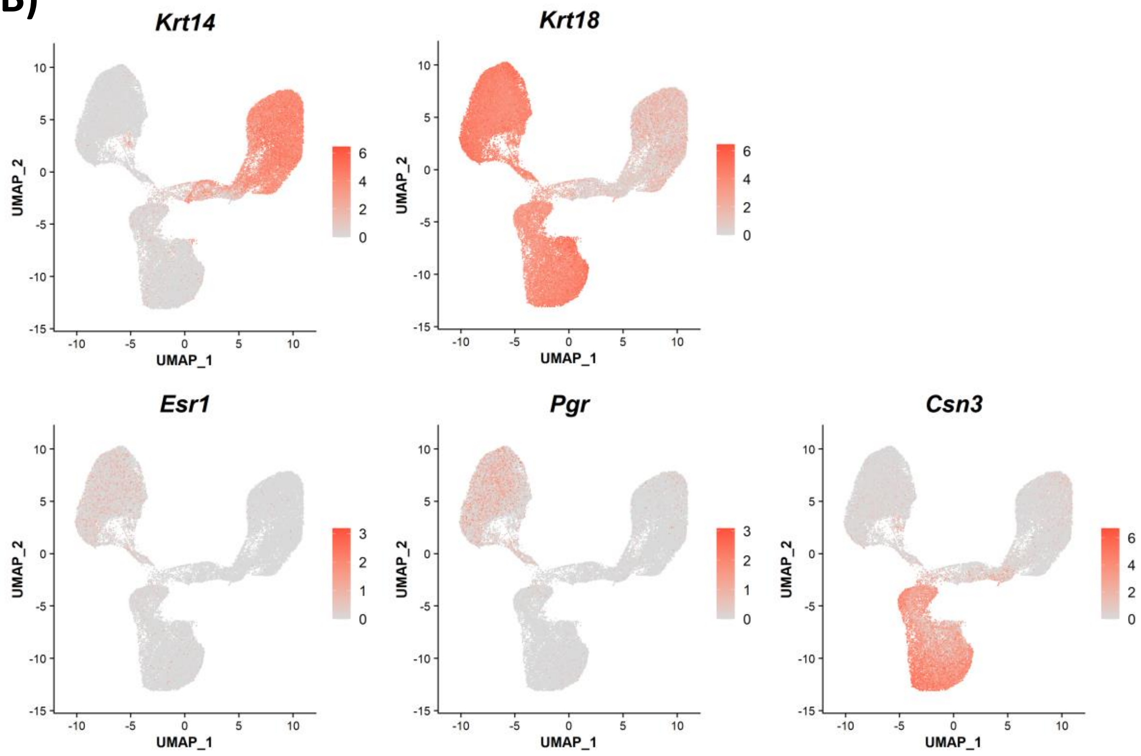

Krt14
Krt18
Esr1
Pgr
Csn3
UMAP_2
UMAP_1

(C)

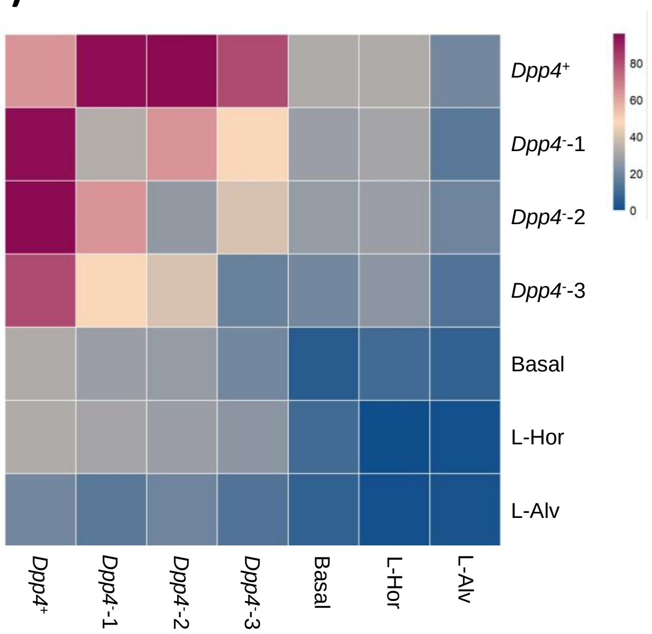

Dpp4+
Dpp4--1
Dpp4--2
Dpp4--3
Basal
L-Hor
L-Alv

Supplementary Figure 5. Initial analysis for the cell-cell interaction inference. **(A)** The UMAP plot of the mammary epithelium dataset. Each cell is represented by each dot (n = 50,408) and is colored according to the cluster identified in our previous paper (Saeki et al., 2021). The basal cells (green), luminal alveolar cells (L-Alv, orange), and luminal hormone-sensing cells (L-Hor, red) were included into the cell-cell interaction inference after the downsampling (n = 20,928). **(B)** The expression of marker genes for each epithelial population identified in our previous publication (*Krt14*, *Krt18*, *Esr1*, *Pgr*, and *Csn3*) (Saeki et al., 2021). Each feature plot shows the distribution of the marker-expressing cells in the mammary gland epithelial cell dataset. Color indicates the expression level. **(C)** The number of ligand-receptor pairs between the mammary gland fibroblasts and the mammary epithelial cells inferred using the CellphoneDB python package. Color of each square indicates the number of the inferred interaction pairs between the cell types indicated in each row and column.

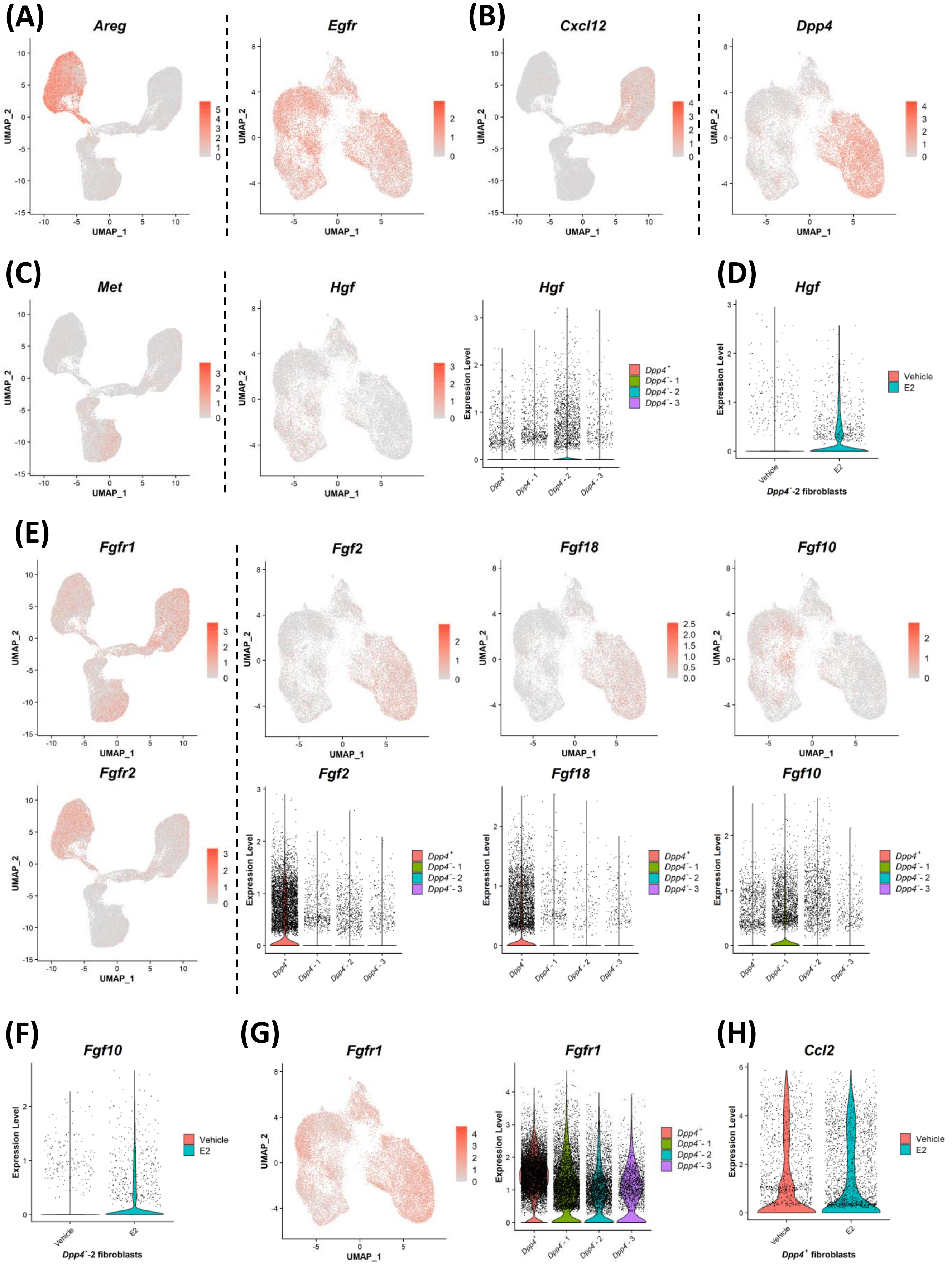

(A)
Areg
Egfr
(B)
Cxcl12
Dpp4
(C)
Met
Hgf
Hgf
(D)
Hgf
Vehicle
E2
Dpp4⁻-2 fibroblasts
(E)
Fgfr1
Fgf2
Fgf18
Fgf10
Fgfr2
Fgf2
Fgf18
Fgf10
Dpp4⁺
Dpp4⁻ - 1
Dpp4⁻ - 2
Dpp4⁻ - 3
(F)
Fgf10
Vehicle
E2
Dpp4⁻-2 fibroblasts
(G)
Fgfr1
Fgfr1
(H)
Ccl2
Vehicle
E2
Dpp4⁺ fibroblasts
UMAP_1
UMAP_2
Expression Level

Supplementary Figure 6. The expression of ligand-receptor pairs identified by the cell-cell interaction inference in the mammary epithelium and fibroblast datasets. **(A)** *Areg* expression in the epithelium and *Egfr* expression in the fibroblasts. **(B)** *Cxcl12* expression in the epithelium and *Dpp4* expression in the fibroblasts. **(C)** *Met* expression in the epithelium and *Hgf* expression in the fibroblasts. **(D)** The expression of *Hgf* in the vehicle- and E2-treated *Dpp4*$^{-}$-2 fibroblasts. **(E)** *Fgfr1*/*Fgfr2* expressions in the epithelium and *Fgf2/Fgf18*/*Fgf10* expressions in the fibroblasts. **(F)** The expression of *Fgf10* in the vehicle- and E2-treated *Dpp4*$^{-}$-2 fibroblasts. **(G)** The expression of *Fgfr1* in the integrated mammary gland fibroblast dataset. **(H)** The expression of *Ccl2* in the vehicle- and E2-treated *Dpp4*$^{+}$ fibroblasts. Each feature plot shows the distribution of the marker-expressing cells in the mammary epithelial cells (**left panels in A-C, and E**) and the mammary gland fibroblasts (**right panels in A-C, and E and G**) with color indicating the expression level. Each violin plot in **(C-H)** shows the expression levels of the genes in each cluster identified in the integrated mammary gland fibroblast dataset (**C, E, and G**) or in each treatment groups (**D, F, and H**).
